# Guanylate-binding protein-1 is a potential new therapeutic target for triple-negative breast cancer

**DOI:** 10.1186/s12885-017-3726-2

**Published:** 2017-11-07

**Authors:** Melissa Quintero, Douglas Adamoski, Larissa Menezes dos Reis, Carolline Fernanda Rodrigues Ascenção, Krishina Ratna Sousa de Oliveira, Kaliandra de Almeida Gonçalves, Marília Meira Dias, Marcelo Falsarella Carazzolle, Sandra Martha Gomes Dias

**Affiliations:** 1 Brazilian Biosciences National Laboratory (LNBio), Brazilian Center for Research in Energy and Materials (CNPEM), Campinas, São Paulo 13083-970 Brazil; 2 Genomic and Expression Laboratory (LGE), Institute of Biology, University of Campinas (UNICAMP), Campinas, São Paulo Brazil; 3 Graduate Program in Genetics and Molecular Biology, Institute of Biology, University of Campinas (UNICAMP), Campinas, São Paulo Brazil

**Keywords:** Breast cancer, Triple-negative breast cancer, Gene expression, RNA-Seq, Transcriptomics, Therapeutic target

## Abstract

**Background:**

Triple-negative breast cancer (TNBC) is characterized by a lack of estrogen and progesterone receptor expression (*ESR* and *PGR*, respectively) and an absence of human epithelial growth factor receptor (*ERBB2*) amplification. Approximately 15–20% of breast malignancies are TNBC. Patients with TNBC often have an unfavorable prognosis. In addition, TNBC represents an important clinical challenge since it does not respond to hormone therapy.

**Methods:**

In this work, we integrated high-throughput mRNA sequencing (RNA-Seq) data from normal and tumor tissues (obtained from The Cancer Genome Atlas, TCGA) and cell lines obtained through in-house sequencing or available from the Gene Expression Omnibus (GEO) to generate a unified list of differentially expressed (DE) genes. Methylome and proteomic data were integrated to our analysis to give further support to our findings. Genes that were overexpressed in TNBC were then curated to retain new potentially druggable targets based on in silico analysis. Knocking-down was used to assess gene importance for TNBC cell proliferation.

**Results:**

Our pipeline analysis generated a list of 243 potential new targets for treating TNBC. We finally demonstrated that knock-down of Guanylate-Binding Protein 1 (*GBP1 *), one of the candidate genes, selectively affected the growth of TNBC cell lines. Moreover, we showed that *GBP1* expression was controlled by epidermal growth factor receptor (EGFR) in breast cancer cell lines.

**Conclusions:**

We propose that GBP1 is a new potential druggable therapeutic target for treating TNBC with enhanced *EGFR* expression.

**Electronic supplementary material:**

The online version of this article (10.1186/s12885-017-3726-2) contains supplementary material, which is available to authorized users.

## Background

The emergence of next-generation sequencing (NGS) technology has provided a large amount of data, much of which is publicly available [1, 2]. Specifically, RNA-Seq has been used for the estimation of RNA abundance [3, 4], alternative splicing detection [5–7], and the discovery of novel genes and transcripts. As such, RNA-Seq has become an important tool in cancer studies [6], contributing to reduced costs and less time being spent in benchtop experiments, thus speeding up the resolution of biological problems. However, a challenge remains in achieving intelligible data analysis and efficient laboratory validation.

Triple-negative breast cancer (TNBC) is characterized by a lack of estrogen and progesterone receptor expression (*ESR* and *PGR*, respectively) and an absence of human epithelial growth factor receptor (*ERBB2*) amplification. Approximately to 15–20% of breast malignancies are TNBC [8]. Patients with TNBC often exhibit unfavorable histopathologic features at diagnosis, mainly consisting of a higher histologic grade, larger tumor size, and frequent metastasis to the lymph nodes [9]. As a consequence, TNBC is associated with a shorter median time to relapse and death [10]. TNBC represents an important clinical challenge since it does not respond to hormone therapy, which targets the abovementioned receptors [11, 12]. Moreover, TNBC is highly heterogeneous [13], indicating the necessity of identifying unifying molecular targets, which may help guide more efficient and less toxic therapeutic management [14, 15].

Guanylate-Binding Protein-1 (*GBP1*) is a member of the large GTPase family and is induced by interferons [16] and inflammatory cytokines [17]. *GBP1* is also transcriptionally regulated by epidermal growth factor receptor (EGFR). In glioblastoma [18, 19] and esophageal squamous cell carcinoma [20], *GBP1* upregulation via the EGFR signaling pathway contributes to tumor proliferation and migration both in vitro and in vivo. Moreover, GBP1 is described as a component of the cytoskeletal gateway of drug resistance in ovarian cancer [21, 22]. *GBP1* expression is also linked to a lack of responsiveness to radiotherapy in some tumors [23], and *GBP1* is overexpressed in pancreatic cancer that is refractory to oncolytic virus therapy [24].

In this work, we utilized RNA-Seq data obtained from TNBC tissues as well as cell lines that were publicly available from The Cancer Genome Project (TCGA) and the Gene Expression Omnibus Portal (GEO), respectively, to search for new therapeutic targets for TNBC. To complement our findings, we also performed transcriptomics analyses of several TNBC cell lines. The obtained lists of overexpressed genes were inter-crossed and compared with data from normal tissues from the TCGA. Methylome and proteomic data were integrated to our analysis to give further support to our findings. Using this approach, we identified 243 genes, which were subsequently evaluated for their druggability potential. *GBP1* was the second gene on the list, and knock-down of *GBP1* in TNBC and non-TNBC cell lines showed that its expression is important for TNBC cell growth. In addition, we demonstrated that *GBP1* expression is controlled by EGFR signaling in breast cancer cells. Thus, we present GBP1 as a new potential druggable target for TNBC with enhanced *EGFR* expression.

## Methods

### RNA sequencing and data processing

Total RNA extraction was performed using the RNeasy kit (Qiagen) according to the manufacturer’s instructions. Then, mRNA was isolated with either the Dynabeads mRNA purification kit (Life Technologies) or the TrueSeq RNA sample preparation kit v2 (Illumina) for samples sequenced at the High-Throughput Sequencing Facility (HTSF) of the University of North Carolina at Chapel Hill (UNC, USA) and the High-Performance Technologies Central Laboratory (LaCTAD) of the University of Campinas (UNICAMP, Brazil), respectively. After isolation, the mRNAs were fragmented in the presence of divalent cations and high temperatures and then employed for cDNA synthesis with random primers using the Superscript II Reverse Transcriptase (Life Technologies) kit. The MDAMB231 and SKBR3 samples were sequenced at HTSF, while the MDAMB436, MDAMB468, BT549 and MCF7 samples were sequenced at LaCTAD. All samples were sequenced using the paired-end × 100 base pairs technique on the Hiseq2000 platform (Illumina). Level 3 TCGA RNA-Seq data (RNASeqV2 raw count estimates) and related clinical data (immunohistochemical results for ER, PR and HER2 TNBC markers) for 1093 tumor tissues from the Breast Invasive Carcinoma (BRCA) dataset, as well as 112 normal breast tissue samples, were downloaded from the Genomic Data Commons Legacy Archive (National Cancer Institute) on November 10, 2016, from *legacy* database. Cell line RNA-Seq data (accession codes GSE58135 [25] and GSE48213 [26]) were obtained from the Gene Expression Omnibus [27] by downloading raw FASTQ files from the DDBJ Sequence Read Archive [28] (DRA) or NCBI Sequence Read Archive (SRA) [29]. FastQC [30] was used to evaluate the quality of the reads. Reads presenting a mean quality score below 30 were removed. Those that exhibited a quality score above this threshold but included bases at the extremities with a quality score below 20 were trimmed using Skewer [31] following guidelines published elsewhere [32], up to a minimum of 30 base pairs. The processed reads were aligned against the hg19 genome using STAR [33], and transcript abundance was estimated with RNA-Seq by Expectation-Maximization (RSEM) [34]. We applied upper-quantile normalization to perform batch effects adjustments and render dataset from distinct sources comparable [35].

### Assignment of breast cancer marker status in the TCGA cohort

The TCGA normalized log2 RSEM values for the *ESR1*, *PGR* and *ERBB2* genes were adjusted to a bimodal curve using an approach published previously [36, 37]. Briefly, for each gene, log_2_ + 1-transformed [38], upper quartile-normalized [35] gene expression was fitted for a 2-component Gaussian mixture distribution model with the R package mclust [39]. The highest match between the assignment and clinical data (when available) was the criterion for selecting equal or variable variance between the two Gaussian fits. For the microarray validation datasets, the same approach was used, but log_2_ + 1-transformed normalized intensity values were used instead.

### Differential gene expression analysis

Differential gene expression analysis of the RNA-Seq data was performed with the R package DESeq2 [40]. The differentially expressed (DE) genes list was restricted to genes showing a fold-change higher or equal than +2 and lower or equal than −2 and a false discovery ratio (FDR) equal to or below 0.05. The microarray datasets were pre-processed using the justRMA function from affy [41], and probes were pooled into genes with Weighted Correlation Network Analysis (WGCNA) [42]. For these data, the DE gene list was generated with limma [43] using eBayes fit. Heatmaps were constructed with the R package heatmap [44] using Pearson’s correlation coefficient and the complete clustering method. Venn plots were constructed with the R package VennDiagram [45], and principal component analysis (PCA) plots were obtained with the R package ggbiplot [46].

### Pathway enrichment, literature annotations and druggability

When possible, GeneIDs or UCSC gene names were translated into Human Genome Organisation (HUGO) annotations using R package org.Hs.eg.db [47]. Gene Ontology [48, 49] annotations were obtained with the R package [50] GO.db [51] (using Wallenius approximation and adjusting *p*-values with the FDR). We employed the R package RISmed [52] to retrieve published papers containing the target gene names and the keyword “triple-negative breast cancer” on November 10, 2016. Interaction network, structural information, structural druggable criteria and druggability rankings was assessed using the canSAR [53] database. Structural drug pockets were assessed using PockDrug [54].

### DNA methylation analysis

The ratio of the methylated probe intensity and the overall intensity (sum of methylated and unmethylated probe intensities), or beta value, were obtained from the HumanMethylation450 BeadChip analysis of the TCGA BRCA samples. The data, downloaded from the Genomic Data Commons Archive (National Cancer Institute) on March 15, 2016, was both quantile normalized and logit transformed using wateRmelon [55]. TNBC, Non-TNBC and normal samples were separated and comparisons at probe-level were performed with limma [43, 56]. The closest transcription initiation site (TSS) and island definition according to the Hidden Markov Models CpG-Islands (HMM CG Islands) [57] were performed with FDb.InfiniumMethylation.hg19 [58]. Shore, shelf and open sea extension of CG Islands was determined with GenomicRanges [59]. Circos plot [60] was performed with OmicCircos [61].

### Proteomics analysis

The Cancer Proteomic Atlas (TCPA) Reverse Phase Protein Array (RPPA) data [62] replicate-based normalized [63] were obtained from the TCPA data portal (http://tcpaportal.org/tcpa/), separated into TNBC, Non-TNBC and normal status and compared with limma [43, 56]. Mass spectrometry normalized and processed data available for the same tumor tissues were obtained from previous work [64]. The limma [43, 56] package was used for the comparisons.

### Cell culture

The triple-negative breast cancer cell lines BT549 (HTB-122™), HCC38 (CRL-2314™), HCC1806 (CRL-2335™), Hs578T (HTB-126™), MDA-MB-157 (HTB-24™), MDA-MB-231 (HTB-26™), MDA-MB-436 (HTB-130™), and MDA-MB-468 (HTB-132™) and the non-triple-negative MCF7 (HTB-22™), SKBR3 (HTB-30™) and T47D (HTB-133™) lines were obtained from the American Type Culture Collection (ATCC) and maintained in RPMI 1640 supplemented with 10% fetal bovine serum and incubated at 37 °C under 5% CO_2_ in a humidified atmosphere.

### Quantitative PCR

RNA samples were extracted with the TRI Reagent (Sigma) following the manufacturer’s instructions. cDNA synthesis was performed using GoScript™ Reverse Transcriptase (Promega) and a 12 μM concentration of a mixture of random hexamers and (dT)18 (7:5), according to the manufacturer’s instructions. PCR amplification was performed with Power SYBR Green PCR MasterMix (Applied Biosystems), as instructed by the manufacturer. Samples were analyzed on the Applied Biosystems 7500 real-time PCR system via the 2^-ΔΔCt^ method [65]. The following primers were used: *rRNA18S * (5′-ATTCCGATAACGAACGAGAC-3′ and 5′-TCACAGACCTGTTATTGCTC-3′), *RPLP0* (5′-GCTCTGGAGAAACTGCTGCCT-3′ and 5′-TGGCACAGTGACTTCACATGG-3′), *GBP1 * (5′-ACTTCAGGAACAGGAGCAAC-3′ and 5′-TATGGTACATGCCTTTCGTC-3′).

### *GBP1* knock-down and in vitro proliferation assay

The pLKO.1-TRC.puro cloning vector (a gift from David Root - Addgene plasmid # 10878) was modified in our laboratory to express the monomeric Kusabira-Orange2 fluorescence protein (mKO2) instead of the selection marker. The shRNA contained the following target sequences: Luc: 5′-CTTACGCTGAGTACTTCGAC-3′; *GBP1*_1: TRCN0000116119 (5′-CGACGAAAGGCATGTACCATA-3′); *GBP1*_2: TRCN0000116120 (5′-TGAGACGACGAAAGGCATGTA -3′). Annealed forward and reverse oligos were cloned into AgeI-EcoRI restriction sites. Viral particle packing was performed, followed by titration, at the LNBio Viral Vector Laboratory Facility. The viruses were transduced at a multiplicity of infection (MOI) of 0.75 with 8 μg/mL of hexadimethrine bromide (Sigma Aldrich, H9268) in 31.25 cells/mm^2^, in triplicate. The medium was replaced after 24 h of transduction and every 48 h thereafter. After 96 and 192 h of transduction, the cells were fixed with 3.7% formaldehyde in 1X phosphate buffered saline (PBS) for 20 min at room temperature and stained with 1.5 μM DAPI (in PBS 1X) for 10 min. Images were collected with an Operetta fluorescence microscope (Perkin Elmer) and analyzed with Columbus (Perkin Elmer). The total number of cells was determined by identifying DAPI-stained nuclei, and positive-for-transduction cells were identified as those exhibiting an mKO2 mean and contrast fluorescence intensity above a threshold defined in non-transduced cells (background signal). The percentage of proliferation (when the number of the cells at time 192 > time 96) as well as the percentage of cell loss (when the number of the cells at time 192 < time 96) were calculated using the following equations: percentage of proliferation: 100*{[shGBP1(Time192/Time96)]/[shLUC(Time192/Time96)]}; cell loss: 100*(1-[shGBP1(Time192/Time96)]). In order to determine GBP1 knockdown long-term effect, we cloned shGBP1 and shLuc sequences into pLKO1-TRC.puro and transduced four cells lines (HCC1806, MDA-MB-436, Hs578T, MDA-MB-231). After a week of puromycin selection, 31.25 cells were seeded per square millimeter into 96 wells plate, and fixed 24 h later (day 1) as described above. Consecutive plates were fixed every 48 h up to 7 days. Number of nuclei was quantified as described above and displayed as the ratio to the number of cells at day 1. Cell cycle phase quantification was determined by DAPI staining as previously described [66].

### Apoptosis assay

Apoptotic/necrotic cells were quantified by Propidium Iodide (PI) staining as previously described [67]. After 7 days of transduction and puromycin selection, cells were collected (both adhered as well as those floating in the media), fixed in 70% ethanol, stained with PI and analyzed by BD FACS Canto II Flow Cytometer with a 488-nm laser line at the FL-3 channel. Control cells were treated with 1 μM Staurosporine to determine the hypodiploid (sub-G1) peak.

### EGFR activation

Cell lines were serum starved for 24 h and then treated with 50 ng/mL of epidermal growth factor (EGF, Sigma-Aldrich) for six hours. *GBP1* expression was quantified via qPCR, and EGFR activation was confirmed by immunoblotting. Cells were washed twice with cold PBS and lysed in lysis buffer (10 mM EDTA pH 8.0, 100 mM Tris-HCl pH 7.4, 150 mM NaCl, 10 mM sodium pyrophosphate, 100 mM NaF, 2 mM PMSF, 10 mM Na_3_VO_4_, 2 μg/ml aprotinin, 10 μM leupeptin, 1 μM pepstatin, 1% Triton X-100). Protein lysates were resolved in 4–20% gradient polyacrylamide SDS gels and transferred onto PVDF membranes via semi-dry electroblotting using six WypAll X60 (Kimberly-Clark) filter pads under alcohol-free buffer conditions [68] at 0.325 mA/mm^2^ for 7 min. The membranes were blocked in 3% non-fat dry milk diluted in Tris Buffered Saline with 0.05% Tween 20, subsequently incubated with anti-p-EGFR (Y1068; Cell Signaling Technology), then washed and probed with HRP-conjugated secondary antibodies (Sigma) for 1 h at room temperature. Band detection was conducted with SuperSignal West Pico Chemiluminescent Substrate (Pierce) followed by autoradiography film exposure.

## Results

### TNBC patient re-classification based on *ESR, PGR and ERBB2* expression data

Since some of the TCGA patients were not classified by immunohistochemistry (IHC) according to Estrogen Receptor (ER), Progesterone Receptor (PR) and Human Epidermal growth factor Receptor 2 (HER2) status (Additional file 1: Figure S1A), we used the corresponding normalized gene (*ESR*, *PGR* and *ERBB2*, respectively) expression levels (determined using a previously proposed approach [36, 37]; Additional file 1: Figure S1B) to define their tissues marker status. For this purpose, the distribution of the expression levels of each gene was fitted with several bimodal mixture possibilities, and the results were compared with the available IHC information (Additional File 1: Figure S1C). The best bimodal model combination achieved 95.3% overall agreement with the available information (Additional File 1: Figure S1D and E) and was used for classification (Additional File 2: Table S1).

### TNBCs exhibit a distinct gene expression pattern

**Fig. 1:**
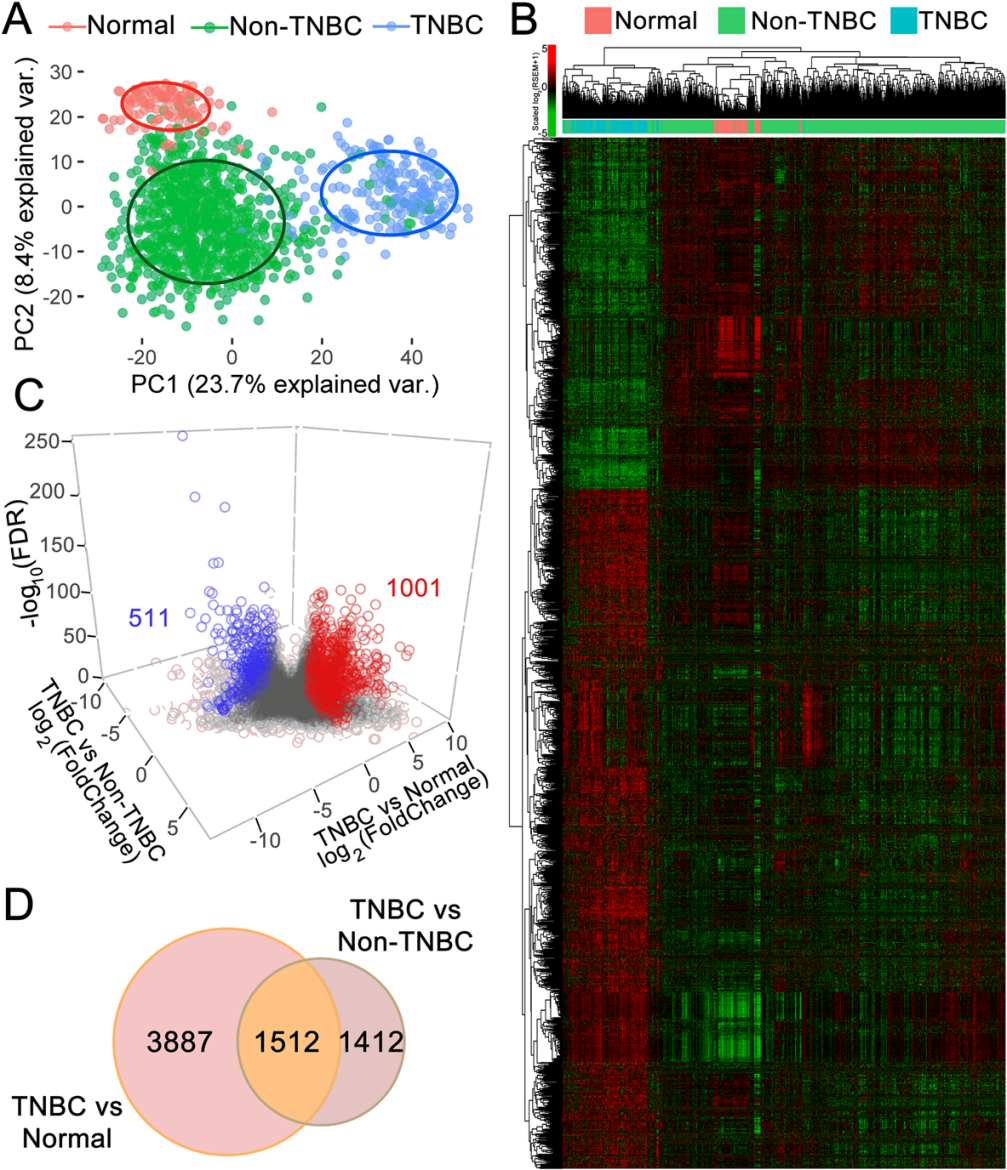
DE genes in TNBC versus non-TNBC tissues and TNBC versus normal tissues from TCGA. Principal component analysis (**a**) and heatmap clustering (**b**) performed with the DE genes revealed a clear separation between TNBC, non-TNBC and normal tissues. Correlations were obtained through Pearson coefficient analysis; unsupervised clustering was conducted via a complete method, and both axis and log_2_(RSEM + 1) values were scaled by line. **c** 3D Volcano plot showing non-DE (gray circles) and DE (blue circles, downregulated; red circles, upregulated) genes. Genes showing FC ≥ +2 and FC ≤ −2 with FDR ≥ 0.05 were considered up- and downregulated, respectively. On axis Z, −log_10_(FDR). **d** Venn diagram showing that 1512 genes were equally DE when TNBC versus non-TNBC and TNBC versus normal tissues were compared

RNA-seq data from 194 TNBC and 899 non-TNBC cases (Additional File 1: Figure S1F and G) were employed to define DE genes using the DESeq2 [40, 69] routine (Additional File 3: Table S2). Similarly, a DE list was generated by comparing TNBC with normal tissues (Additional File 4: Table S3). A total of 2924 DE genes were identified when TNBC was compared with non-TNBC, while 5399 DE genes were identified between TNBC and normal tissues (Additional File 5: Figure S2A and B, respectively). The DE list efficiently separated both pairs of groups, as denoted by unsupervised (Fig. 1a) and supervised (Additional File 5: Figure S2C and D) PCA. The same trend was observed when a hierarchical clustering analysis was conducted (Fig. 1b). Curiously, TNBC tissues presented greater spatial separation for both components in the comparison with normal tissues versus the comparison with non-TNBC tumors, as further demonstrated by exclusive clustering. A total of 1512 DE genes were shared between the two lists, with 1001 genes being upregulated (fold-change (FC) ≥ +2) and 511 being downregulated (FC ≤ −2), with a FDR equal to or less than 0.05 (Fig. 1c and d and Additional File 6: Table S4).

### TNBC cell lines are good surrogates for studying the disease

With the aim of using cell lines to validate the new targets, we first compared the gene expression profiles of the cell lines with tumor tissues. To this end, we sequenced four TNBC (MDA-MB-231, BT549, MDA-MB-436 and MDA-MB-468) and two non-TNBC cell lines (MCF7 and SKBR3; data processing with Skewer [31], shown in Additional File 7: Figure S3A), referred to as “in-house” cell lines herein. The RNA-Seq results were confirmed by comparing the expression levels of 48 genes (displayed as log_2_ RSEM + 1) with the data obtained through qPCR (1/ΔC_t_). The obtained Spearman correlations varied between 0.40 (MCF7) and 0.67 (SKBR3) (Additional File 7: Figure S3B). To complement our analysis, we added the RNA-Seq data from other six TNBC cell lines (MDA-MB-157, Hs578T, HCC70, HCC1806, HCC1937 and HCC1143) and two non-TNBC cell lines (T47D and ZR75–1), which were available from GEO (see Additional file 8, Table S5, for a description of all presented data). All 14 cell lines were rendered comparable after proper normalization, despite variations in the applied sequencing methods (Additional File 7: Figure S3C). We first confirmed the TNBC status of the cell lines by verifying *ESR1*, *PGR1* and *ERBB2* expression levels (Additional File 9: Figure S4). A total of 4033 DE genes were identified between the TNBC and non-TNBC cell lines, with 2300 being upregulated and 1733 being downregulated (Additional File 10: Figure S5A; Additional File 11: Table S6). As observed in the patient tissue data, unsupervised PCA clearly separated TNBC from non-TNBC cell lines (Additional File 10: Figure S5B), which was confirmed through hierarchical clustering (Additional File 10: Figure S5C).

**Fig. 2:**
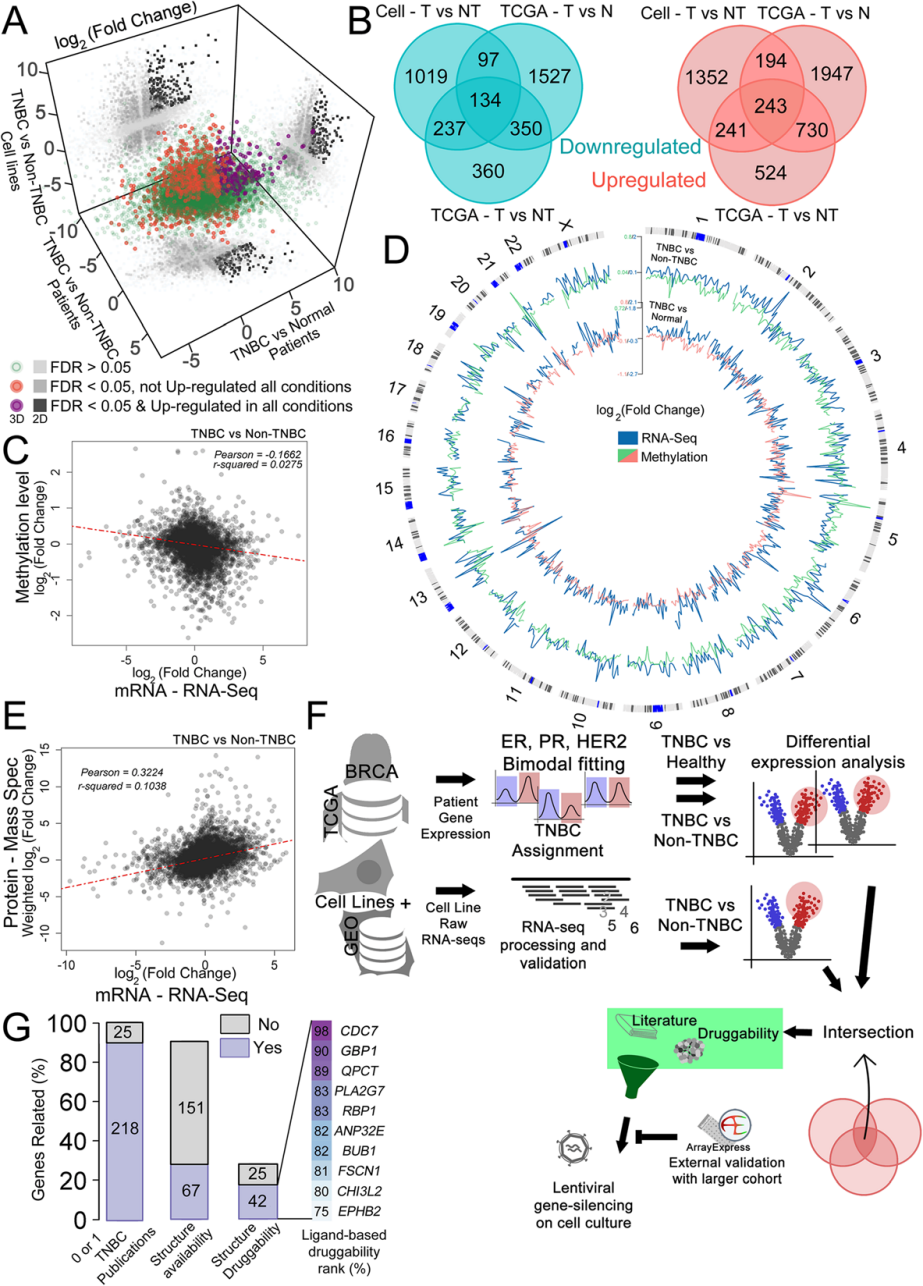
Transcriptomics and proteomics druggability analysis generated a list of new potential protein targets for TNBC. **a** 3D correlation plot between FC of DE genes. Dark gray in 2D projections represents upregulated genes. Unifying DE genes exhibiting an FDR ≤ 0.05 and an FC ≥ +2; FDR ≤ 0.05 and FC ≤ −2; or an FDR > 0.05 are shown as purple, orange and green circles, respectively. **b** Venn diagrams showing that 134 genes (B, left) were equally downregulated, while 243 (**b**, right) were equally upregulated in all three comparisons. **c** Probes covering CpG islands were related to genes based on TSS proximity and their methylation status (values for different probes were averaged) were correlated to the gene expression FC (TNBC x Non-TNBC). **d** Circos plot comparing CpG islands methylation FC (green or pink lines) with gene expression FC (blue line) in the TNBC x Non-TNBC (outer circle) or TNBC x normal (inner circle) (chromosome ideogram denoted in the most outer circle). Values for both methylation and gene expression FC were averaged within every 5 Mbp. FC opposite spikes indicate that the higher the methylation FC, the lower the gene expression FC of the associated region, and vice-verse. **e** Protein level FC (MS dataset [64] performed with the same BRCA samples used in this work) and gene expression FC correlation in the comparison TNBC x Non-TNBC. **f** Pipeline used for new protein targets discovering. **g** Number of genes found in two or more publications (25) or in 0 or 1 publication (218) following the PubMed query “gene name + triple-negative breast cancer”. The genes that were non-cited or were cited only once were then evaluated in canSAR as either having available protein structure (67) or not (151), followed by a cutoff of being structurally druggable (42) or not (25). Among the 42 genes with a druggable structure, the top 10 based on the ligand-based druggability percentile are listed

By crossing the TNBC and non-TNBC DE gene lists obtained from the tissue and cell line analyses with the list of DE genes obtained in the comparison of TNBC versus normal tissues (Tri-dimensional plot in Fig. 2a; Two-dimensional view in Additional File 12: Figure S6; Gene list in Additional File 13: Table S7), we identified 134 common downregulated and 243 common upregulated genes (Fig. 2b). Curiously, pairwise correlations between fold-changes revealed a positive Pearson correlation of 0.35 in the comparison of TNBC vs. non-TNBC tissues with TNBC vs. non-TNBC cell lines (Additional File 12: Figure S6, most right), indicating agreement in the overall differential expression profiles. We then performed Gene Ontology (GO) analysis to verify whether the two types of samples exhibited common enriched biological processes, molecular functions and cellular components. Several of these processes and pathways were equally enriched in TNBC versus non-TNBC in both tissues and cell lines (Additional File 14: Figure S7). Considering our results together, we conclude that TNBC cells are distinct from normal tissues, which creates an interesting window for searching for therapeutic targets. Moreover, established cell lines retain a high resemblance to tumor tissues, making them good surrogates for testing potential new targets for treating TNBC.

### CpG methylation status of potential regulatory regions concur with expression level of DE genes

Aside from the transcriptomic and genomic information available from the TCGA, the project also make available methylation and proteomic (Reverse Phase Protein Array, RPPA) data for most of the samples found in the platform. DNA methylation is the most-studied epigenetic modification in mammalian cells and is characterized by the addition of a methyl group at the carbon-5 position of cytosine residues within CpG dinucleotides. Intrigued whether there was or not a correlation between the methylation status of CpG islands with the gene expression FC variation found in the TNBC versus non-TNC and TNBC versus normal comparisons, we crossed the transcriptomic with the methylome data. To do so, DNA methylation data (Additional File 15: Figure S8A) was quantile normalized (Additional File 15: Figure S8B), logit transformed (Additional File 15: Figure S8C) and differentially methylated regions (DMR) defined in TNBC versus Non-TNBC and TNBC versus Normal tissue (Additional File 15: Figure S8D-E, Additional file 16: Table S8). Within the generated list of hypermethylated (FC ≥ +2) and hypomethylated (FC ≤ −2) regions found in the TNBC samples (in comparison to non-TNBC or normal samples) is a region already described for the *PPFIA3* gene [70]. Similarly, we found the islands cg10029842 and cg17473600 (chr1–47,207; exon of LHX8) as hypermethylated in TNBC samples, as already described [70]. Hypermethylation (as opposed to hypomethylation) of both islands are related to lower survival time in TNBC patients [70]. Of note, we observed more hypomethylated (than hypermethylated) probes in TNBC, concurring with previous publications [71].

DMRs may be present at CpG islands (regions larger than 200pb in length with >50% GC content), shores (up to 2 kb from CpG islands), shelves (2-4 kb from CpG islands) and open-sea (isolated CpG in the genome) [72]. CpG islands placed at regions nearby to transcriptional start sites (TSS), when hypermethylated, are highly likely to cause gene downregulation, the opposite also being true [73].

When we analyzed only probes covering CpG islands, associated them to genes based on TSS proximity and related their methylation FC with the gene expression FC obtained from the TNBC x Non-TNBC comparison, we found a negative Pearson correlation of ~ − 0.17 (Fig. 2c). This data indicates that promoter region hypermethylation may partially explain the alteration in the expression level (the higher the methylation status, the lower the mRNA level) seen in the TNBC x Non-TNBC comparison. Coherence between higher gene expression level and lower methylation status (as well as the other way around) can be overall appreciated in the Circos plot of the Fig. 2d. We concluded that alteration on the expression level status of the TNBC tissues (compared to Non-TNBC) can be partially explained by the methylation level of CpG islands placed nearby to the TSS of these genes.

### TNBC x non-TNBC gene expression fold change overall agrees with protein level fold change

Higher or lower gene expression levels do not do not necessarily correlate to protein levels. We used the RPPA data to calculate protein FC in TNBC (compared to Non-TNBC and normal tissues). Then, we compared the protein FC with the gene expression FC of the TNBC versus Non-TNBC and TNBC versus normal tissues comparisons and found a Pearson correlation of 0.73 (Additional file 17: Figure S9A) and 0.46 (Additional file 17: Figure S9B), respectively. In parallel, we used mass spectrometry (MS) data available for the same BRCA group of patients used in our gene expression analysis [64] to evaluate the correlation between gene expression and protein level FC in TNBC versus Non-TNBC. Equally to the comparison performed with the RPPA data, the MS comparison displayed a positive Pearson correlation of 0.32 (Fig. 2E and Additional file 17: Figure S9C). In summary, we found a positive correlation amongst gene expression and protein level FC in the evaluated gene lists.

### Common overexpressed genes and druggability criteria used to reveal new potential targets for TNBC

Using all of the gathered information, we created a pipeline for selecting new targets (Fig. 2f). To do so, we took a closer look at the list of overexpressed genes. For 10% of the genes, there were at least two published papers linking them to TNBC (Fig. 2g). The remaining 90% were then evaluated with the canSAR platform to search for druggable targets. canSAR is an integrated knowledge base that combines data on biology, pharmacology, structural biology, cellular networks and clinical annotations to provide druggability predictions [74]. Out of the remaining 218 targets, 67 had available structure information, 42 of which presented structure-based druggability (Fig. 2g), as they showed potential small molecule binding pockets in an analysis based on the ChEMBL Strudel https://www.ebi.ac.uk/chembl/drugebility/) (DrugEBIlity) methodology. Among these genes, 10 exhibited ligand-based druggability scores falling within the 75% percentile or above defined for all of the proteins in the platform (Fig. 2g). This parameter is an easy way to assess how a target’s druggability compares with that of all other targets in the proteome and aims to estimate the likely druggability of a target based on the chemical properties and bioactivity parameters of small molecule compounds (including molecular weight, med-chem friendliness and ligand-efficiency) that have been tested against the protein itself and/or its homologues. If the target binds drug-like compounds, it is more likely to be druggable than a target that only binds compounds with very un-drug-like properties.

### Guanylate-binding protein 1 (*GBP1*) is more expressed in TNBC

**Fig. 3:**
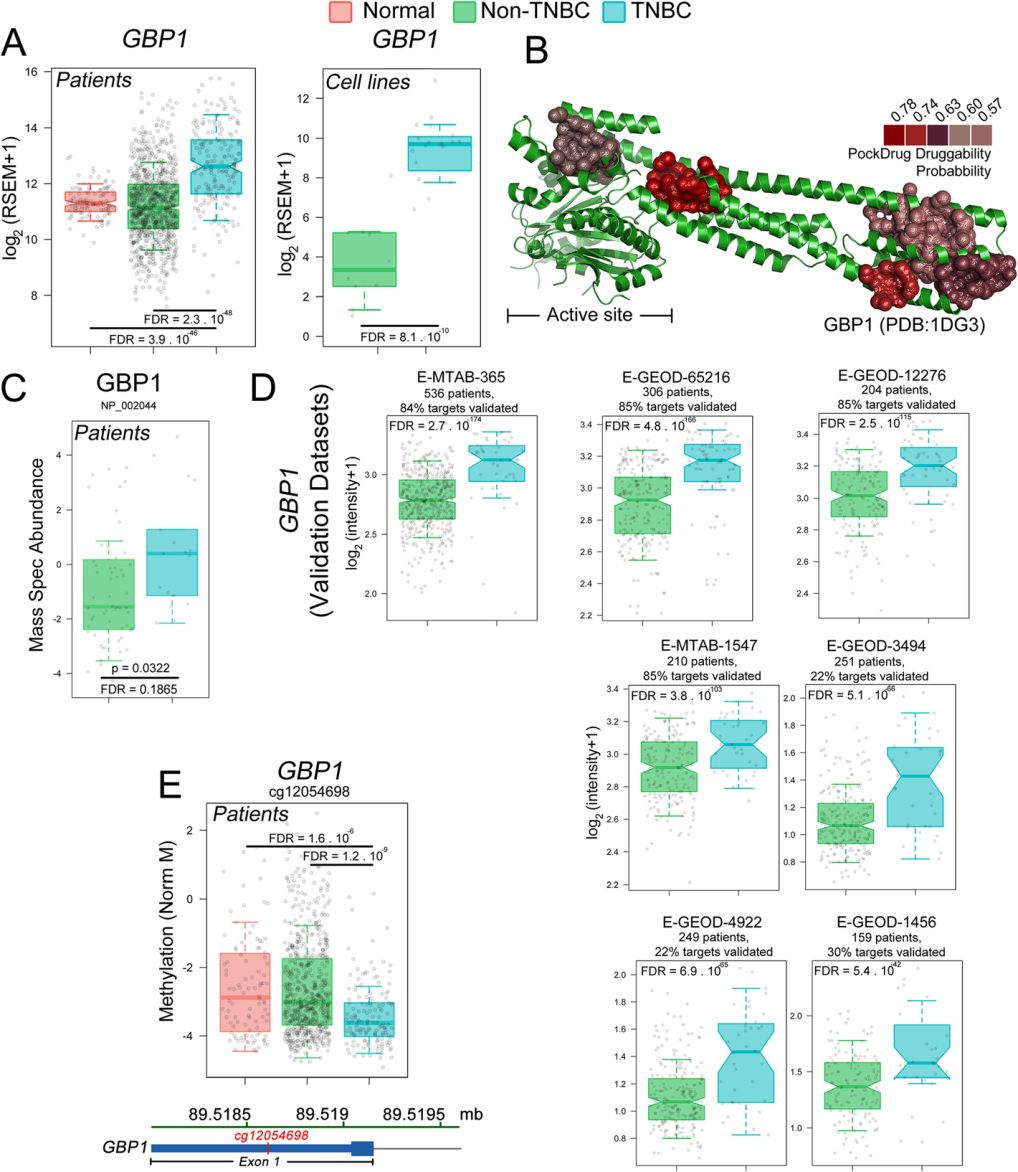
Multiple evidence sources makes GBP1 arise as potential target for TNBC. **a** *GBP1* is more highly expressed in TNBC than in non-TNBC and normal tissues (left) and in TNBC versus non-TNBC cell lines (right). FDR values were obtained from the DESeq2 comparisons. **b** Cartoon representation of the human GBP1 protein structure (PDB ID 1DG3), displaying the 5 highest-scoring potential small molecule binding pockets according to PockDrug [54]. **c** MS evaluation of GBP1 protein level in Non-TNBC and TNBC samples. *P*-Value and FDR value were calculated with limma. **d** Seven microarray datasets external-to-our-pipeline analysis confirmed *GBP1* upregulation in TNBC versus non-TNBC tissues. FDR values were derived from limma comparisons. (**e**, lower) GBP1 gene scheme denoting the open-sea probe cg12054698 location within the exon 1. (**e**, upper) Methylation status (as defined by M-values) for the cg12054698 in Normal, Non-TNBC and TNBC samples, showing hypomethylation in TNBC. FDR values calculated with limma. As for all the displayed box-plots, log_2_-transformed upper-quantile values were used, with the whiskers extending to half of the interquartile range. Gray circles denote each sample. Notches, when present, denotes the 95% confidence intervals of the median

Cell Division Cycle 7 (*CDC7*), the first in the final top 10 list, has recently been described as a therapeutic target to treat TNBC [75, 76]. *GBP1* was listed second in the final list of potential druggable targets. *GBP1* is a member of an interferon-inducible gene family, the p65 guanylate-binding proteins (GBPs). GBPs are structurally related to the dynamins and another known antiviral protein family, the Mx proteins. *GBP1* is clearly overexpressed in TNBC tissues (Fig. 3a, left) and cell lines (Fig. 3a, right) and has at least 5 possible binding pockets for drug interactions (Fig. 3b) as calculated by PockDrug [54]. Moreover, GBP1 protein level is also enhanced in TNBC compared to non-TNBC samples as evaluated by MS protein analysis (Fig. 3c). The preferentially higher expression of *GBP1* in TNBC tissues versus non-TNBC tissues was further confirmed in 7 other microarray datasets (totaling 1915 patients; Fig. 3d), confirming *GBP1* as a potential new druggable target for this disease. All of the datasets were processed following the same approach used for the TCGA datasets (Additional File 18: Figure S10A). Our final list of overexpressed genes was finally crosschecked with the lists of overexpressed genes obtained from these 7 external microarray datasets, revealing intersections varying from 22% to 85% (Additional File 18: Figure S10B and C). Finally, by looking at the *GBP1* methylation status, we found an open-sea DMR in the 5′ UTR region of the gene (Fig. 3e, lower scheme), which is hypomethylated in TNBC samples when compared to normal and Non-TNBC samples (Fig. 3e). This finding provides potential regulatory mechanism behind GBP1 higher expression level on TNBC.

In order to access the impact of GBP1 expression on the disease prognosis, we used the Nearest Centroid Classifier for Area Under Curve optimization (NCC-AUC) model [77] to integrate patient 5-years survival status with RNA expression level. By using a λ of 10^−5^ and θ-score cutoff of 10^−5^, the analysis showed that ~17% of our final gene target list would be potential targets based on the impact of their expression level on patient survival, which did not include *GBP1* (Additional file 19: Table S9). Indeed, we verified that there is no difference on *GBP1* expression level in patients with less than 5 years survival time (Additional file 20: Figure S11) compared to patients with more than 5 years survival time (*p* = 0.49). Altogether, our data show that *GBP1* is more expressed (and is also present at higher protein level) in TNBC, which may be related to hypomethylation of a CpG open-sea region present at the 5’UTR. *GBP1* higher expression did not affect TNBC patient prognosis.

### Guanylate-binding protein 1 (*GBP1*) knock-down exclusively affects TNBC cell growth

**Fig. 4:**
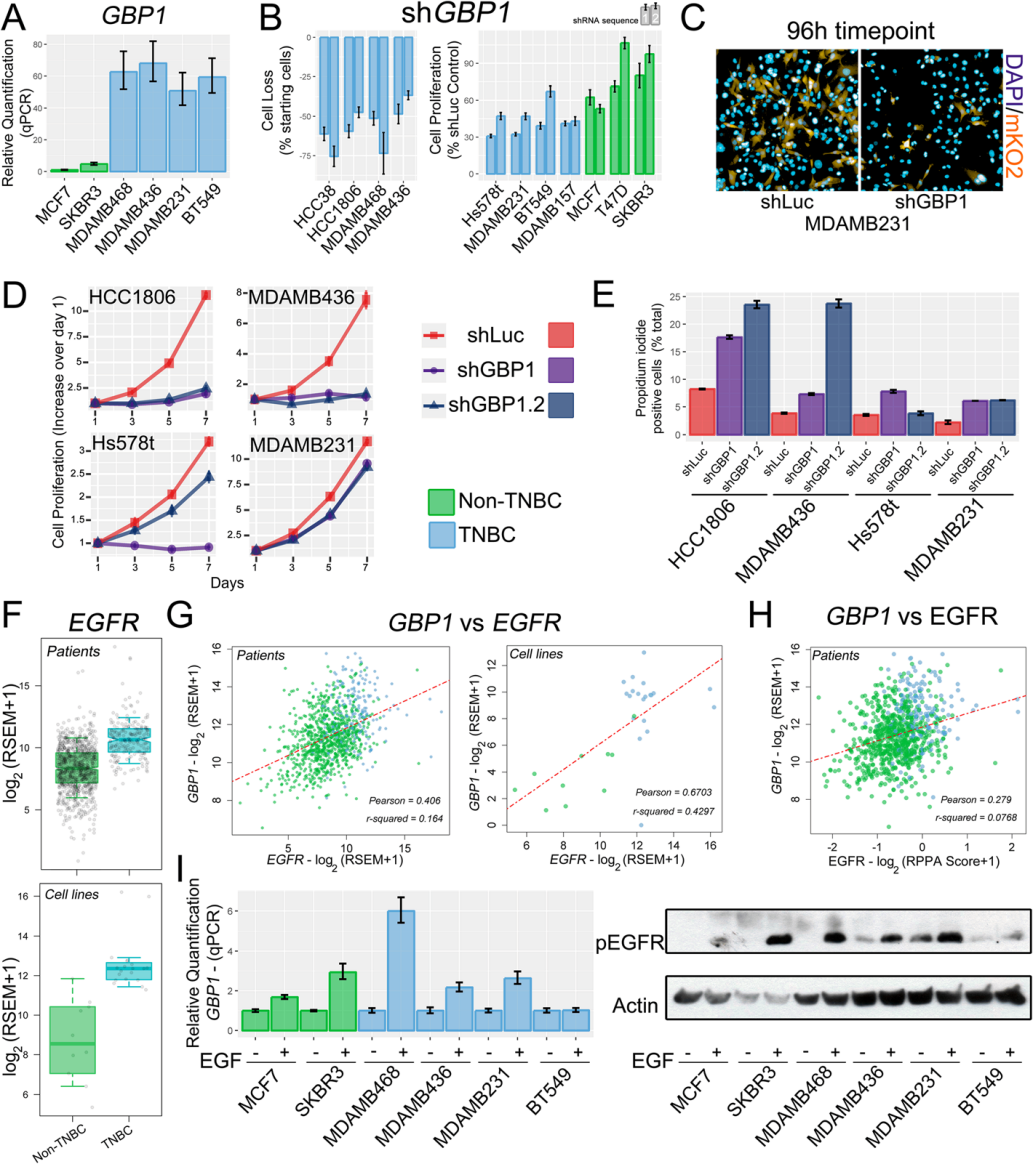
TNBC are more sensitive to *GBP1* knock-down than non-TNBC cells. EGFR drives *GBP1* expression. **a** *GBP1* mRNA levels were evaluated via quantitative PCR in different cell lines. **b** *GBP1* knock-down (sh*GBP1*) using *pLKO.mKO2* for 96 h affected the growth of TNBC cells more effectively than that of non-TNBC cells, as assessed using two shRNA sequences. An shRNA targeting non-human gene luciferase (shLuc) was used as a control. Data were split between cells that died (left) and cells that proliferated less (right) after knock down. **c** Representative fluorescence microscopy images of MDA-MB-231 after 96 h of *GBP1* knock-down compared with shLuc. DAPI staining of nuclei is shown in blue, and mKO2 fluorescence of cells positive for viral transduction is shown in yellow. **d** Cell proliferation assay (performed over 7 days) of cell lines selected to stably express the shGBP1 and shLuc sequences. **e** Propidum iodide incorporation assay was performed to evaluate the fraction of cells that are in apoptosis/late necrosis state. EGFR is more highly expressed in TNBC than non-TNBC tissues (**f**, top) and cell lines (**f**, down). The FDR value was absent in DESeq2 comparisons due to outlier removal. **g** *GBP1* and *EGFR * expression levels are highly correlated in tissues (left) and cell lines (right). **h** *GBP1* expression level positively correlates with EGFR total protein level. Log_2_-transformed upper-quantile RSEM expression values were used, with whiskers extending to half of the interquartile range. Gray circles denote each sample Notches denote the 95% confidence interval of the median. (I) MDA-MB-231 cells were serum starved for 24 h and then stimulated with 50 ng/mL of EGF for six hours. Western blotting (right) confirmed that the treatment increased EGFR stimulation (increase of Tyr1068 phosphorylation). qPCR (left) showed that, with the exception of BT549, all tested cell lines responded to EGF stimulation by increasing *GBP1* expression. Error bars denote one standard error of the experimental triplicates

Having shown that TNBC cell lines are good surrogates for studying the disease, we next confirmed that GBP1 is more highly expressed in TNBC cell lines than in non-TNBC cell lines via qPCR (Fig. 4a). We then tested the importance of *GBP1* for TNBC cell proliferation compared with non-TNBC cells. We assayed eight TNBC and three non-TNBC cell lines by knocking-down *GBP1* with two different shRNA sequences (with knock-down efficiencies of 68% and 81% as assessed via qPCR, Additional File 21: Figure S12A) and using a sequence targeting the Luciferase gene (Luc) as a negative control. Overall, knocking-down *GBP1* with either of the shRNA sequences resulted in more profound effects on the proliferation of TNBC cells than non-TNBC cells (Fig. 4b and c). To evaluate long-term impact of *GBP1* knock down on cells that responded either dying (HCC1806 and MDS-MB-436) or proliferating less (Hs578t and MDA-MB-231) after GBP1 knock down, we transduced cell lines and selected them to stably express the shRNA sequences. After checking the knocking down efficiency of the transduced cell lines (Additional File 21: Figure S12B), we evaluated cell proliferation for 7 days. The data showed that, while Hs578t and MDA-MB-231 maintained the slower proliferation behavior seeing on the endpoint assay (with the exception of the shRNA #1 tested on Hs578t), HCC1806 and MDA-MB-436 selected cells had their growth profoundly affected by the knock down (Fig. 4D), likely because of the increased rate of cell death seeing for these cells (Fig. 4e and Additional File 21: Figure S12C). Accordingly, MDA-MB-231 cells expressing the sh*GBP1* #1 and #2, compared to control shLuc, present a slight (but significative) percentage increase of cells in the G0-G1 phase, and a slight but significative percentage decrease of cells in the S phase, indicating cell growth arrest at the G0-G1 phase (Additional File 21: Figure S12D-E). HCC1806 cells responded on the opposite direction (Additional File 21: Figure S12D-E). In summary, we demonstrated that *GBP1* is overexpressed and important for the survival of a subgroup of TNBC cells.

### GBP1 interaction network

To provide information on the functional connection of GBP1 with other cellular proteins, we performed an interaction network analyzes as implemented by the canSAR platform. GBP1 either physically interact (directly or indirectly) or is functionally related to several proteins (Additional File 22: Figure S13). *GBP1* expression is induced by Interferon Regulatory factors (IRF) 2, 3 and 9, coherent with the GBP1 being a member of an interferon-inducible family [78]. GBP1 is also a transcriptional target of the STAT 1 (which acts as a heterodimer with STAT2), a downstream effector of the interferon signaling pathway [79]. The Protein arginine N-methyltransferase 1 (PRMT1) methylates arginine residues of several proteins, including histones. GBP1 arginine methylation functionally connects PRMT1 to GBP1. Interferon-stimulated gene 15 (ISG15), a protein that adds itself covalently to other proteins (in a process similar to ubiquitination), was shown to physically interact with GBP1 [80]. Finally, the Specificity protein 1 (SP1), a transcriptional factor that controls many different cellular process, also binds to GBP1 [81]. FNTA and FNTB are both subunits of the farnesyltransferase and the geranylgeranyltransferase complexes, which transfer a farnesyl or geranylgeranyl moieties to proteins, affecting their function. In summary, network interaction analysis performed by canSAR highlight the already known interplay of GBP1 with the interferon signaling pathway and implicate that disturbing GBP1 function in cells have the potential to impact such pathway. It also reveals binding partners related to diverse functions in the cells and may point to some yet unexplored roles of GBP1.

### EGFR drives *GBP1* expression in breast cancer

EGFR is one of the major biomarkers of TNBC, predicting a poor outcome of the disease [82], and it has been reported as a new target for treating TNBC [83]. As expected, *EGFR* was found to be overexpressed in TCGA TNBC tissues compared with expression in non-TNBC tissues (Fig. 4f, upper panel), in the in-house sequenced and GEO cell lines (Fig. 4f, lower panel). RPPA analysis also confirmed that EGFR protein level is enhanced in TNBC compared to non-TNBC (Additional File 23: Figure S14). EGFR is known to control *GBP1* expression in glioblastoma and esophageal carcinoma [18–20], and (not surprisingly) we verified a positive correlation between *EGFR* and *GBP1* expression levels when TNBC and non-TNBC tissues (Pearson correlation coefficient = 0.41; Fig. 4g, left panel) and cell lines (Pearson correlation coefficient = 0.67; Fig. 4g, right panel) were compared. We also compared EGFR protein levels (according to the RPPA data) with *GBP1* expression levels, obtaining a Pearson correlation coefficient of 0.28 (Fig. 4h). Furthermore, we confirmed the EGFR-signaling-dependent expression of GBP1 in breast cancer cell lines via qPCR (Fig. 4i). A positive correlation was not observed when we compared *GBP1* and Y1173 or Y1068 EGFR phosphorylation levels in the patient tissue samples (data not shown) using the RPPA data. We conclude that EGFR controls *GBP1* expression in breast cancer cells.

## Discussion

Several works have used transcriptomic analysis to improve the classification of TNBC and to obtain new predictive markers and therapeutic targets for the disease [25, 26, 37, 84–87]. In our approach, we integrated RNA-Seq data from normal and tumor tissues (obtained from TCGA) and from cell lines that were sequenced in-house or were available from the GEO databank. A unifying DE gene list was obtained from the comparisons of normal x TNBC tissues, TNBC x non-TNBC tissues and TNBC x non-TNBC cell lines. Methylome and proteomic data were integrated to our analysis to give further support to our findings. A total of 243 genes were shown to be exclusively overexpressed in TNBC tissues and established cell lines and, importantly, were more highly expressed in TNBC than in non-transformed breast epithelial tissues. Subsequently, we searched for novelty by removing genes that have already been strongly linked to TNBC by analyzing publications listed in PubMed. Finally, we subjected our list to druggability scoring using the multidisciplinary canSAR platform. With the canSAR platform, we were able to predict gene products that could be used as therapeutic targets based on protein structure availability, the presence of potential small molecule binding pockets and information regarding the pre-existence of bio-active compounds (drugs or chemical probes) that have already been tested on a target or its homologues. Thus, we combined transcriptomic and proteomic approaches to enhance our chances of identifying proteins with true potential to become new therapeutic targets.

Moreover, by comparing the GO signatures of the cell lines and tissue transcriptomic data, we showed that cell lines could serve as good surrogates for testing these potential new targets, and we used them to show that *GBP1* (the second highest ranked gene on the final list) knock-down selectively affected TNBC cell growth. *GBP1* expression is controlled by EGFR in glioblastoma [18, 19] and esophageal squamous head and neck cancers [20] and is important for proliferation and tumor invasion. In addition, GBP1 is linked to radiotherapy resistance in head and neck tumors [23] and is a component of the cytoskeletal gateway of drug resistance in ovarian cancer [21, 22], especially for paclitaxel, which is a common therapeutic choice for treating TNBC [88]. Class III β-tubulin plays an important role in the development of drug resistance to paclitaxel by allowing the incorporation of GBP1 into microtubules. Upon entering the cytoskeleton, GBP1 binds to pro-survival kinases, such as Proto-oncogene Serine/threonine-protein kinase pim-1 (PIM1), and initiates a signaling pathway that induces resistance to paclitaxel [89]. Indeed, a 4-aza podophyllotoxin derivative was demonstrated to act as a potent in vitro inhibitor of the GBP1:PIM1 interaction, which is a property that is maintained in vivo in ovarian cancer cells resistant to paclitaxel [90]. Taken together, these findings confirm GBP1 as a druggable protein.

It is well known that the TNBC is a very heterogeneous breast cancer subtype [91]. In saying so, it was not out of surprise that the tested TN cell lines responded heterogeneously to the *GBP1* knock down: Out of the 8 tested cell lines, while 4 presented increased cell death, 4 responded by decreasing cell proliferation in comparison to control. Moreover, *GBP1* expression level did not impact on patient’s 5 years survival as evaluated by the NCC-AUC model. Indeed, we observed that the cell lines that were more impacted by *GBP1* knock down are, following a molecular sub-classification of the disease [37], Basal-like 1 (BL1) and 2 (BL2) cells (with the exception of MDA-MB-436). On the other hand, the cell lines that had only its proliferation affected after *GBP1* knock down are, all of them, mesenchymal (M) or mesenchymal stem-like subtypes (MSL) [37]. Top gene ontologies for the BL1 and BL2 subtype are heavily enriched in cell cycle and cell division components and pathways, as well as growth factor signaling. Differently, the M and MSL subtype display gene ontologies that are heavily enriched in components and pathways involved in cell motility, ECM receptor interaction, and cell differentiation pathways. The MSL, in particular, presents enrichment of genes associated with stem cells and mesenchymal stem cell–specific markers, and low expression of claudins [37]. We hypothesize that higher expression levels of *GBP1* may have a more severe impact on the survival of a subgroup of TNBC patients with specific molecular markers.

*EGFR* is overexpressed in a high proportion of the TNBC cases [82, 92] and is a marker of a poor prognosis [93–95]. Although EGFR has been successfully used as a therapeutic target for many tumor types [96], unencouraging results have been obtained in clinical trials (in both mono- and adjuvant therapy protocols) conducted in TNBC patients [97]. Failure to induce inhibition of Akt has been reported as a major cause of resistance to EGFR inhibitors [97, 98]. Moreover, nuclear EGFR (nEGFR) can enhance resistance to anti-EGFR therapies and correlates with poor overall survival in breast cancer. Inhibition of nEGFR nuclear translocation leads to subsequent accumulation of EGFR on the plasma membrane, which greatly enhances the sensitivity of TNBC cells to cetuximab [99]. We demonstrated that *GBP1* expression correlates with *EGFR* expression (and protein levels) in both tissues and breast cancer cell lines. In most of the tested cell lines, we showed that the *GBP1* expression level responded to EGFR stimulation by epidermal growth factor.

## Conclusions

TNBC is an aggressive histological breast cancer subtype with limited treatment options and very poor prognosis following progression after standard chemotherapeutic regimens. For that, finding new therapeutic targets to fight this disease is of great importance. In this work, by using a combination of transcriptomics and proteomics analysis, we generated a list of 243 potential new therapeutic targets for treating TNBC. Second on this list, we show that *GBP1* expression correlates with EGFR stimulation and is important for TNBC cell proliferation. In summary, we propose that GBP1 is a new potential druggable therapeutic target for treating TNBC with enhanced *EGFR* expression.

## Additional files

Additional file 1: Figure S1. Assignment of breast cancer marker status according to TCGA using RNA expression levels. (A) Number of samples positive for ER, PR and Her2, as determined via IHC and available from the TCGA. In more than 30% of the tissues, at least one of the markers was not classified. (B) Density graph of the raw log2 + 1 transformed RSEM of all genes in the 1100 samples RNA-Seq dataset, showing that the maximum density values largely deviated around an RSEM of 10 (left). Normalization performed with upper-quantile [35] methodology harmonized all of the datasets (right). (C) mClust [39] was used to fit bimodal distribution patterns and define samples that were positive or negative for the expression of *ESR*, *PGR* and *ERBB2*. To do so, some assumptions were made and tested to search for the best combination of assumptions based on the percentage of agreement with the available IHC data. “E” denotes “equal variance between populations”, and “V” denotes “variable variance between populations”. (D) Concordance between expression (using the EEE combination) and IHC data for each marker as well as for all three combined. (E) Bimodal fits, as implemented by mClust with the EEE combination, highlighting samples that are negative (purple) and positive (light pink) for *ESR1* (left), *PGR* (middle) and *ERBB2* (right). (F) Boxplots of the log2-transformed upper-quantile RSEM of the *ESR*, *PGR* and *ERBB2* markers in normal, non-TNBC and TNBC tissues. The whiskers extend to half of the interquartile range. Gray circles denote each sample. Notches denote the 95% confidence interval of the median. (G) Assignment of marker status assignment based on RNA expression levels (PNG 7247 kb)

Additional file 2: Table S1. *ESR1*, *PGR* and *ERBB2* RSEM values of each tumor tissue, marker status according to mclust model and respective available IHC data (PNG 1377 kb)

Additional file 3: Table S2. DE genes between TNBC and non-TNBC tissues (XLS 279 kb)

Additional file 4: Table S3. DE genes between TNBC and normal tissues (XLS 2099 kb)

Additional file 5: Figure S2. Analysis of TNBC versus non-TNBC and TNBC versus normal DE genes from the TCGA samples. Volcano plot of the FC of the genes TNBC versus non-TNBC (A) and TNBC versus normal (B) comparisons. Non-DE (or DE but with a *p*-value >0.05) genes are indicated with gray circles, while DE genes are indicated with blue circles when downregulated and red circles when upregulated. Genes showing an FC ≥ +2 and FC ≤ −2, with an FDR ≥ 0.05, were considered up- and downregulated, respectively. The numbers outside the circles refer to all genes that passed the FDR cutoff, while the numbers inside the circles are DE genes that passed both the FDR and fold-change cutoffs. Principal component analysis using DE genes obtained from TNBC versus non-TNBC (C) and TNBC versus normal tissues (D) comparisons. PCA correlations are denoted with circles around the samples (XLS 2112 kb)

Additional file 6: Table S4. Intersection between DE genes from TNBC x non-TNBC and TNBC x normal tissue (PNG 4690 kb)

Additional file 7: Figure S3. Quality assessment of RNA-Seq data. (A) FastQC [30] plot of the Phred scores of each nucleotide position of all reads before and after Skewer [31] trimming for BT549, MCF7, MDAMB436 and MDAMB468, sequenced at LaCTAD-UNICAMP, and MDAMB231 and SKBR3, sequenced at HTSF-UNC. (B) RNA-Seq data from in-house-sequenced cell lines were evaluated for reproducibility by comparing the log2 RSEM +1 values of 48 genes with the obtained qPCR 1/ΔCT values. Density of raw log_2_-transformed RSEM values for the in-house-sequenced and Varley et al. [25] and Daemen et al. [26] datasets (C, left) and the normalized RSEMs (C, right), showing success in the harmonization of all data, despite variations in sample preparation and sequencing (XLS 4164 kb)

Additional file 8: Table S5. Description of all cell lines (*in house* sequenced or obtained from GEO) used in this work (PNG 2237 kb)

Additional file 9: Figure S4. Cell lines exhibit the expected *ESR*, *PGR* and *ERBB2* marker expression status. Linear range of RSEM from *ESR1* (upper), *PGR1* (middle) and *ERBB2* (lower) for the *in house* and external RNA-Seq datasets. Gray boxes below data indicate the study related to the dataset (*in house*, Varley et al. [25] and Daemen et al. [26]) (XLS 26 kb)

Additional file 10: Figure S5. Analysis of DE genes in TNBC versus non-TNBC cell lines. (A) Volcano plot of the FC and *p*-values of the genes. Non-DE (or DE but with p-value >0.05) genes are shown with gray circles, and DE genes are shown with blue circles when downregulated and red circles when upregulated. Genes with an FC ≥ +2 and an FC ≤ −2, with an FDR ≥ 0.05, were considered up- and downregulated, respectively. The numbers outside of circles refer to all genes that passed the FDR cutoff, while the numbers inside of circles are DE genes that passed both the FDR and fold-change cutoffs. Principal component analysis (B) and correlation heatmap (C) using DE genes obtained from the comparison. PCA correlations are denoted with circles around the samples. Heatmap correlations were obtained through Pearson coefficient analysis; unsupervised clustering was conducted via the complete method, and both axis and log_2_(RSEM + 1) values were scaled by line (PNG 1166 kb)

Additional file 11: Table S6. DE genes between TNBC and non-TNBC cell lines (PNG 6547 kb)

Additional file 12: Figure S6. Correlation plots. 2D correlation plots (equivalent to the 2D projections in Fig. 2A) of the FC of DE genes obtained from the comparisons of TNBC versus non-TNBC cell lines and TNBC versus normal tissues (left), TNBC versus normal tissues and TNBC versus non-TNBC tissues (middle), and TNBC versus non-TNBC cell lines and TNBC versus non-TNBC tissues (right) (PNG 1580 kb)

Additional file 13: Table S7. Intersection between DE genes from TNBC x non-TNBC tissue, TNBC x normal tissue and TNBC x non-TNBC cell lines (XLS 4979 kb)

Additional file 14: Figure S7. GO analysis of altered pathways in TNBC tissues and cell lines (compared with non-TNBC samples). Biological processes (blue), cellular components (red) and molecular functions (green) equally enriched in TNBC tissues (left) and cell lines (right). These pathways point to events occurring on the membrane, associated with signaling activity and cell motility. Each box denotes 1 order of magnitude of FDR reduction; dashed black lines highlight an FDR = 0.05 (PNG 1759 kb)

Additional file 15: Figure S8. Analysis of Infinium HumanMethylation450 BeadChip methylation array from 876 TCGA samples, including TNBC, Non-TNBC and normal tissues. (A) Raw Kernel density plot from β methylation ratios (methylated / total signal) for each category (TNBC, Non-TNBC or normal tissue). (B) Quantile normalized Kernel density plot of β methylation ratios, as implemented by wateRmelon [55]. (C) Quantile normalized Kernel density plot of M-Values (Logit transformation of β normalized ratios), showing the peaks alignment. Volcano plot of the FC and adjusted p-values of the methylation probes in TNBC vs Non-TNBC (D) and TNBC vs normal (E) comparisons. Non-differentially methylated (DM) probes (or differentially methylated one but with p-value >0.05) are shown as gray circles. DM probes are shown as blue circles when hypermethylated and red circles when hypomethylated. Probes with a FC ≥ +2 or an FC ≤ −2, with an FDR < 0.05, were considered hyper- and hypomethylated, respectively. The numbers outside of circles refer to all probes that passed the FDR cutoff, while the numbers inside of circles are DM probes that passed both the FDR and fold-change cutoffs (XLSX 45499 kb)

Additional file 16: Table S8. Methylation status of the available probes in the TCGA TNBC x Non-TNBC and normal x TNBC comparisons, as performed by limma (PNG 2076 kb)

Additional file 17: Figure S9. Proteomic analysis of BRCA tissues by using the RPPA and MS data. (A) Comparison between protein level FC (available from RPPA) and mRNA level FC in TNBC vs Non-TNBC (A) and TNBC vs normal (B) comparisons. RPPA data are limited to only 160 proteins. (C) Volcano plot of the FC versus adjusted *p* values of proteins from MS dataset [64] in TNBC vs Non-TNBC comparison. Non-DE (or DE but with *p*-value > 0.05) proteins are shown as gray circles, and DE proteins are shown as blue circles when down-regulated and red circles when up-regulated. Proteins with an FC ≥ +2 and an FC ≤ -2, with an FDR < 0.05, were considered up- and down-regulated, respectively. The numbers outside of circles refer to all proteins that passed the FDR cutoff, while the numbers inside of the circles are DE proteins that passed both the FDR and fold-change cutoffs. (PNG 4403 kb)

Additional file 18: Figure S10. Evaluation of external Array Express datasets. (A) E-MTAB-365, E-GEOD-65216, E-GEOD-12276, E-MTAB-1547, E-GEOD-3494, E-GEOD-4922 and E-GEOD-1456 expression profiles of ESR1, PGR and ERBB2 and bimodal adjustment (green for samples with lower expression and blue for samples with higher expression). (B) Number of tissues negative (pink) and positive (purple) for each marker as well as for all three markers. (C) Venn diagrams between our identified target list and the upregulated genes identified through TNBC versus non-TNBC analysis of all external datasets. At the intersection of the smaller (genes from our list) and larger circles (upregulated genes from the external datasets), the numbers and percentages of genes in agreement are provided, positioned upwards (XLS 47 kb)

Additional file 19: Table S9. List of genes which expression level impact patients 5 years survival following NCC-AUC analysis (PNG 585 kb)

Additional file 20: Figure S11. Expression level of *GBP1* in patients divided by survival time (more than 5 years survival or less than 5 years survival). The whiskers extend to half of the interquartile range. Gray circles denote each sample. Notches denote the 95% confidence interval of the median. *P*-Value from Welch’s t-test (PNG 1379 kb)

Additional file 21: Figure S12. *GBP1* knock down evaluation and its effect on cell cycle. (A) qPCR of MDA-MB-231 after *GBP1* knock-down, as performed in the end-point assay. (B) qPCR of HCC1806, MDA-MB-231, Hs578t and MDA-MB-231 cells transduced and selected with puromycin to stably express the shRNA sequences. Cell Cycle analysis using DNA content evaluation (as determined by DAPI intensity staining) was executed after imaging attached cells by microscopy. Cells were classified being at the SubG1 (C), G0-G1 (D) or (E) S phase. Error bars represents standard error of the mean. *P*-Values from Welch’s t-tests (PNG 1679 kb)

Additional file 22: Figure S13. GBP1 Interaction network as defined with the canSAR platform (PNG 724 kb)

Additional file 23: Figure S14. Boxplot of RPPA EGFR protein level comparing Non-TNBC with TNBC samples. The whiskers extend to half of the interquartile range. Gray circles denote each sample. Notches denote the 95% confidence interval of the median (PNG 724 kb)

## Abbreviations

ATCC: American Type Culture Collection
BL1: Basal-like 1
BL2: Basal-like 2
BRCA: Breast Invasive Carcinoma
DE: Differentially expressed
DRA: DDBJ Sequence Read Archive
EDTA: Ethylenediamine tetraacetic acid
EGF: Epidermal Growth Factor
EGFR: Epidermal Growth Factor Receptor
ER: Estrogen receptor protein
ERBB2: epithelial growth factor receptor gene
ESR: Estrogen receptor gene
FC: fold change
FDR: false discovery ratio
GBP1: Guanylate-Binding Protein 1
GBPs: guanylate-binding proteins
GEO: Gene Expression Omnibus
GO: Gene Ontology
HER2: Epidermal growth factor Receptor 2 protein
HTSF: High-Throughput Sequencing Facility
HUGO: Human Genome Organisation
IHC: immunohistochemistry
LaCTAD: High-Performance Technologies Central Laboratory
Luc: luciferase
M: Mesenchymal
mKO2: monomeric Kusabira-Orange 2 fluorescence protein
MOI: multiplicity of infection
MSL: Mesenchymal stem-like
Na_3_VO_4_: Sodium orthovanadate
NaCl: Sodium chloride
NaF: Sodium fluoride
NCC-AUC: Nearest Centriod Classifier for Area Under the Curve
nEGFR: nuclear Epidermal Growth Factor Receptor
NGS: Next-generation Sequencing
PBS: phosphate buffered saline
PCA: principal component analysis
PCR: Polymerase chain reaction
PGR: progesterone receptor gene
PIM1: Proto-oncogene serine/threonine-protein kinase pim-1
PMSF: Phenylmethanesulfonyl fluoride
PR: Progesterone receptor protein
PVDF: Polyvinylidene difluoride
RNA-Seq: mRNA sequencing
RPPA: Reverse phase protein array
RSEM: RNA-Seq by Expectation-Maximization
SDS: Sodium dodecyl sulfate
SRA: NCBI Sequence Read Archive
TCGA: The Cancer Genome Atlas
TNBC: Triple-Negative Breast Cancer
Tris-HCl: Tris(hydroxymethyl)aminomethane hydrochloride
UCSC: University of California, Santa Cruz
UNC: University of North Carolina at Chapel Hill
UNICAMP: University of Campinas
WGCNA: Weighted Correlation Network Analysis
