# Supplementary figures and images for: Guanylate-binding protein-1 is a potential new therapeutic target for triple-negative breast cancer
Source: BMC Cancer. 2017 Nov 7;17:727. doi: 10.1186/s12885-017-3726-2 (PMC5688804; doi:10.1186/s12885-017-3726-2)

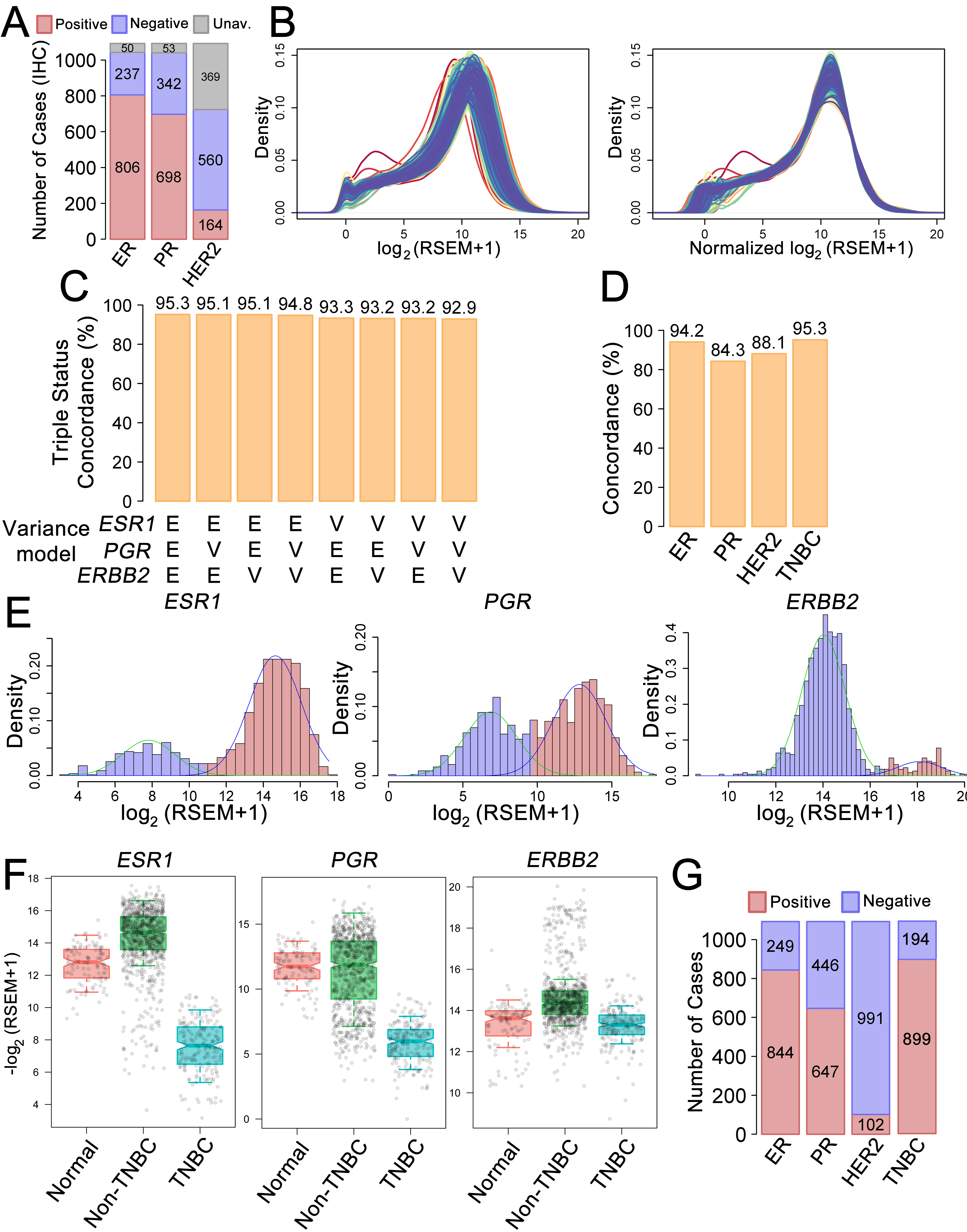

Supplement: Supplementary file 1 — Assignment of breast cancer marker status according to TCGA using RNA expression levels. (A) Number of samples positive for ER, PR and Her2, as determined via IHC and available from the TCGA. In more than 30% of the tissues, at least one of the markers was not classified. (B) Density graph of the raw log2 + 1 transformed RSEM of all genes in the 1100 samples RNA-Seq dataset, showing that the maximum density values largely deviated around an RSEM of 10 (left). Normalization performed with upper-quantile [35] methodology harmonized all of the datasets (right). (C) mClust [39] was used to fit bimodal distribution patterns and define samples that were positive or negative for the expression of ESR, PGR and ERBB2. To do so, some assumptions were made and tested to search for the best combination of assumptions based on the percentage of agreement with the available IHC data. “E” denotes “equal variance between populations”, and “V” denotes “variable variance between populations”. (D) Concordance between expression (using the EEE combination) and IHC data for each marker as well as for all three combined. (E) Bimodal fits, as implemented by mClust with the EEE combination, highlighting samples that are negative (purple) and positive (light pink) for ESR1 (left), PGR (middle) and ERBB2 (right). (F) Boxplots of the log2-transformed upper-quantile RSEM of the ESR, PGR and ERBB2 markers in normal, non-TNBC and TNBC tissues. The whiskers extend to half of the interquartile range. Gray circles denote each sample. Notches denote the 95% confidence interval of the median. (G) Assignment of marker status assignment based on RNA expression levels (PNG 7247 kb) [file 12885_2017_3726_MOESM1_ESM.png]

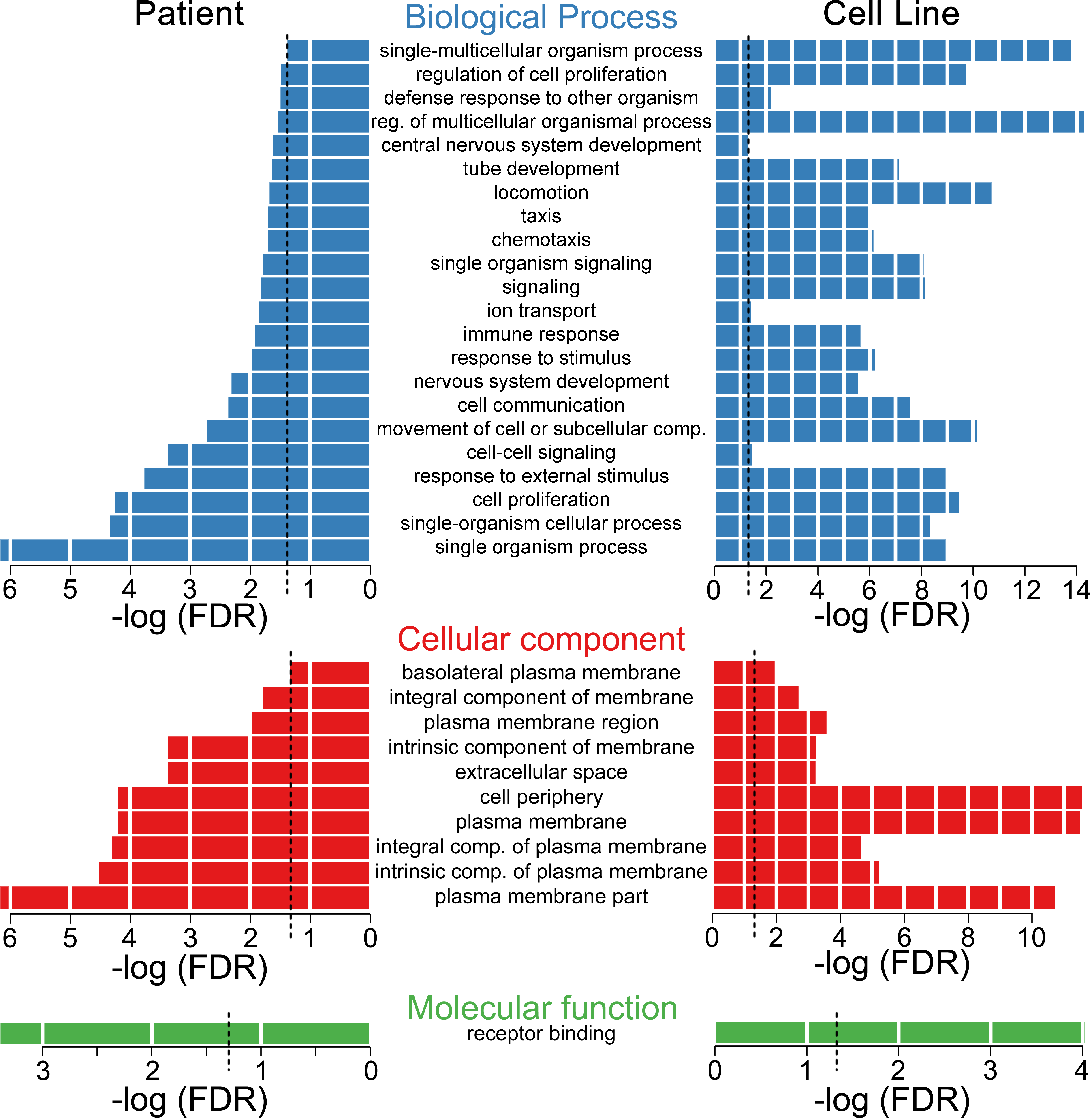

Supplement: Supplementary file 2 — ESR1, PGR and ERBB2 RSEM values of each tumor tissue, marker status according to mclust model and respective available IHC data (PNG 1377 kb) [file 12885_2017_3726_MOESM2_ESM.png]

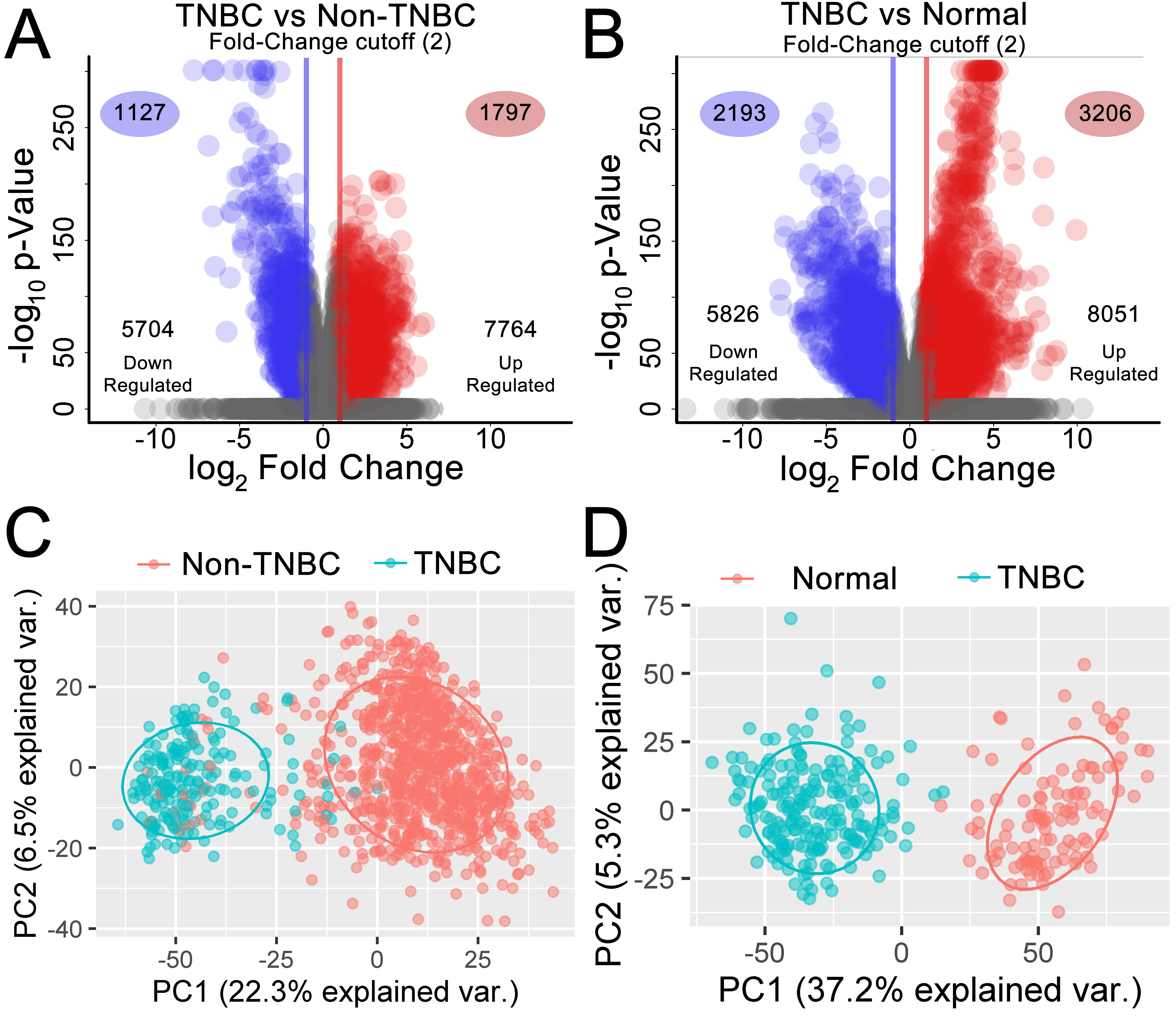

Supplement: Supplementary file 6 — Intersection between DE genes from TNBC x non-TNBC and TNBC x normal tissue (PNG 4690 kb) [file 12885_2017_3726_MOESM6_ESM.png]

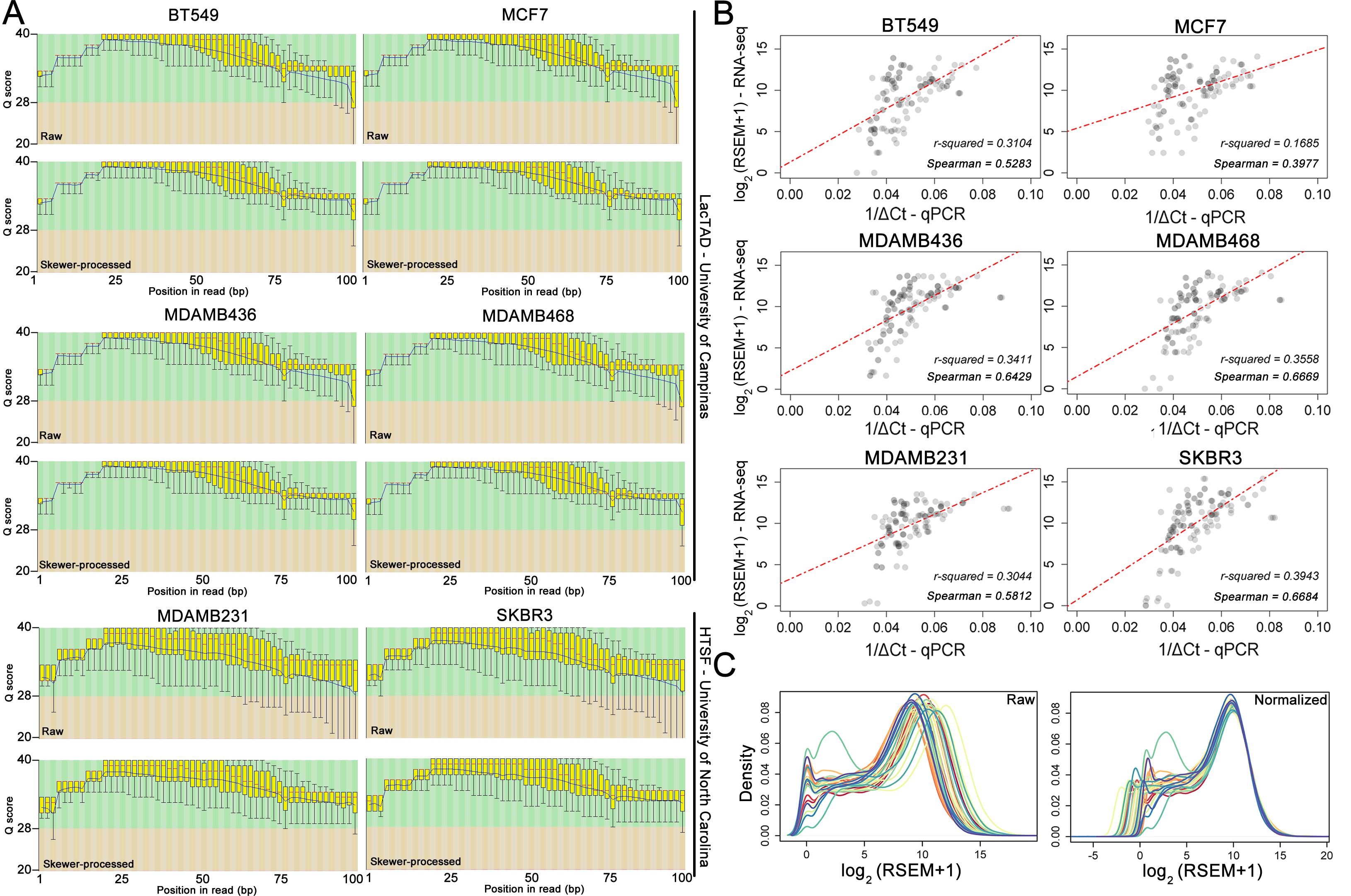

Supplement: Supplementary file 8 — Description of all cell lines (in house sequenced or obtained from GEO) used in this work (PNG 2237 kb) [file 12885_2017_3726_MOESM8_ESM.png]

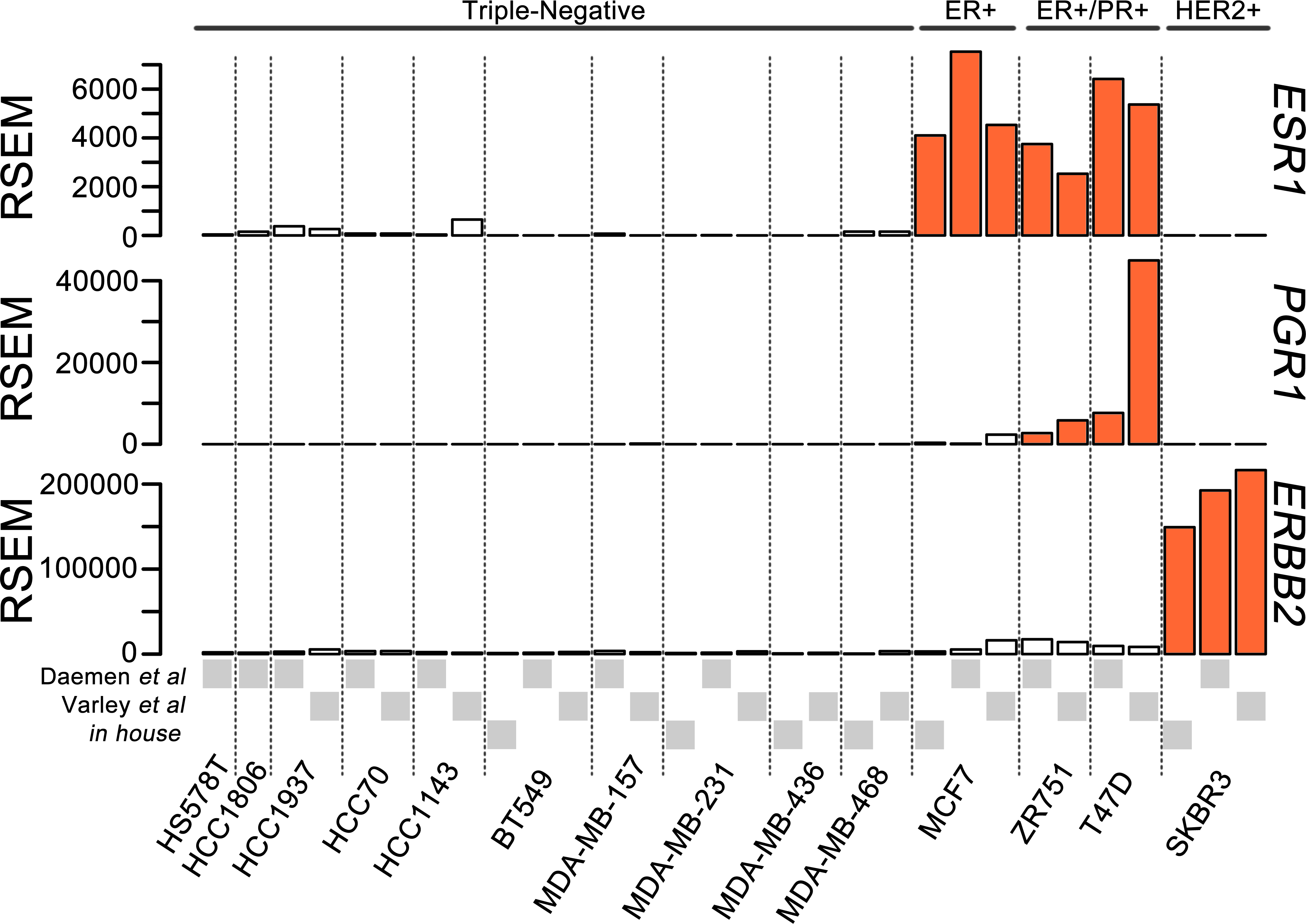

Supplement: Supplementary file 10 — Analysis of DE genes in TNBC versus non-TNBC cell lines. (A) Volcano plot of the FC and p-values of the genes. Non-DE (or DE but with p-value >0.05) genes are shown with gray circles, and DE genes are shown with blue circles when downregulated and red circles when upregulated. Genes with an FC ≥ +2 and an FC ≤ −2, with an FDR ≥ 0.05, were considered up- and downregulated, respectively. The numbers outside of circles refer to all genes that passed the FDR cutoff, while the numbers inside of circles are DE genes that passed both the FDR and fold-change cutoffs. Principal component analysis (B) and correlation heatmap (C) using DE genes obtained from the comparison. PCA correlations are denoted with circles around the samples. Heatmap correlations were obtained through Pearson coefficient analysis; unsupervised clustering was conducted via the complete method, and both axis and log2(RSEM + 1) values were scaled by line (PNG 1166 kb) [file 12885_2017_3726_MOESM10_ESM.png]

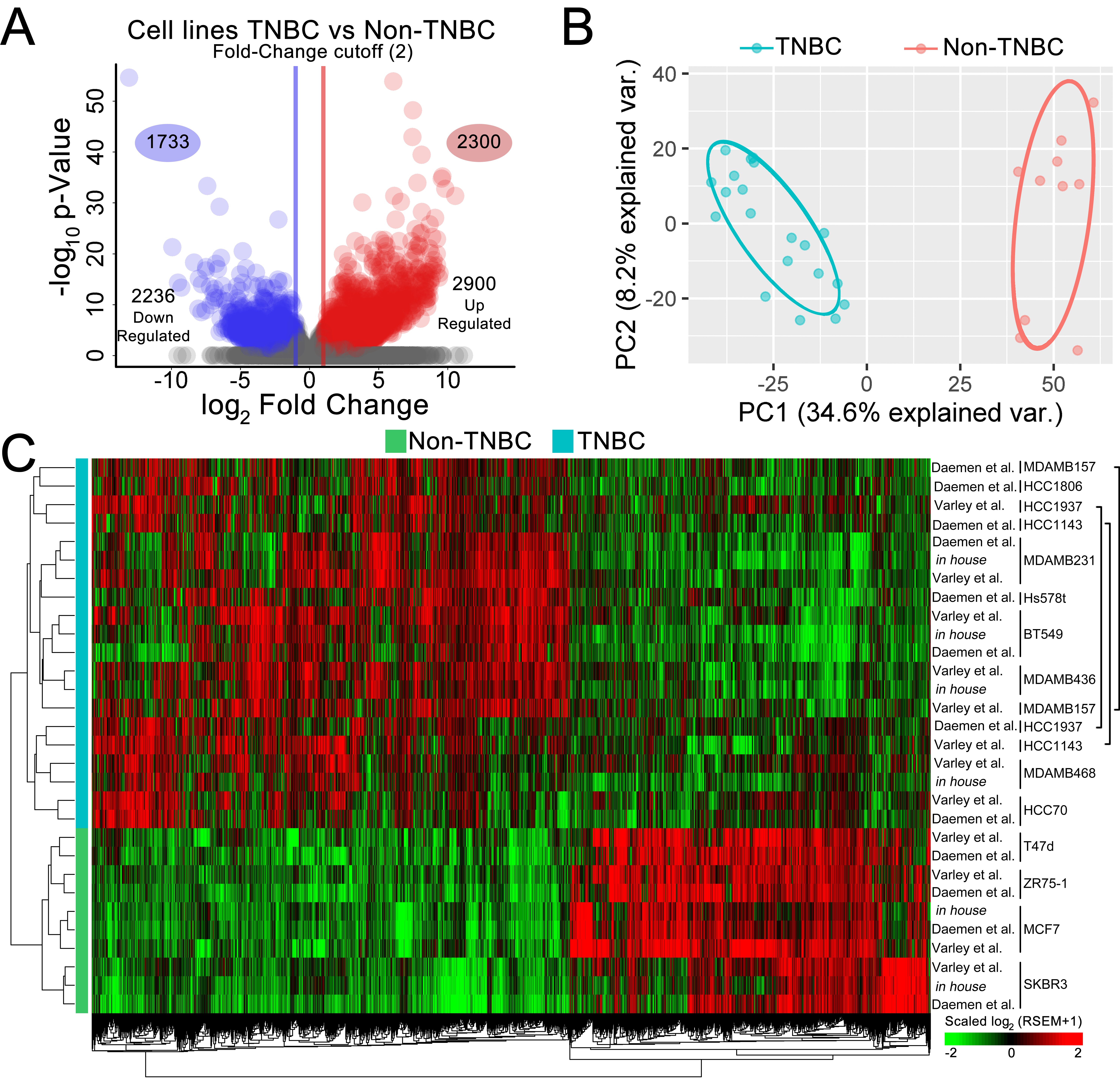

Supplement: Supplementary file 11 — DE genes between TNBC and non-TNBC cell lines (PNG 6547 kb) [file 12885_2017_3726_MOESM11_ESM.png]

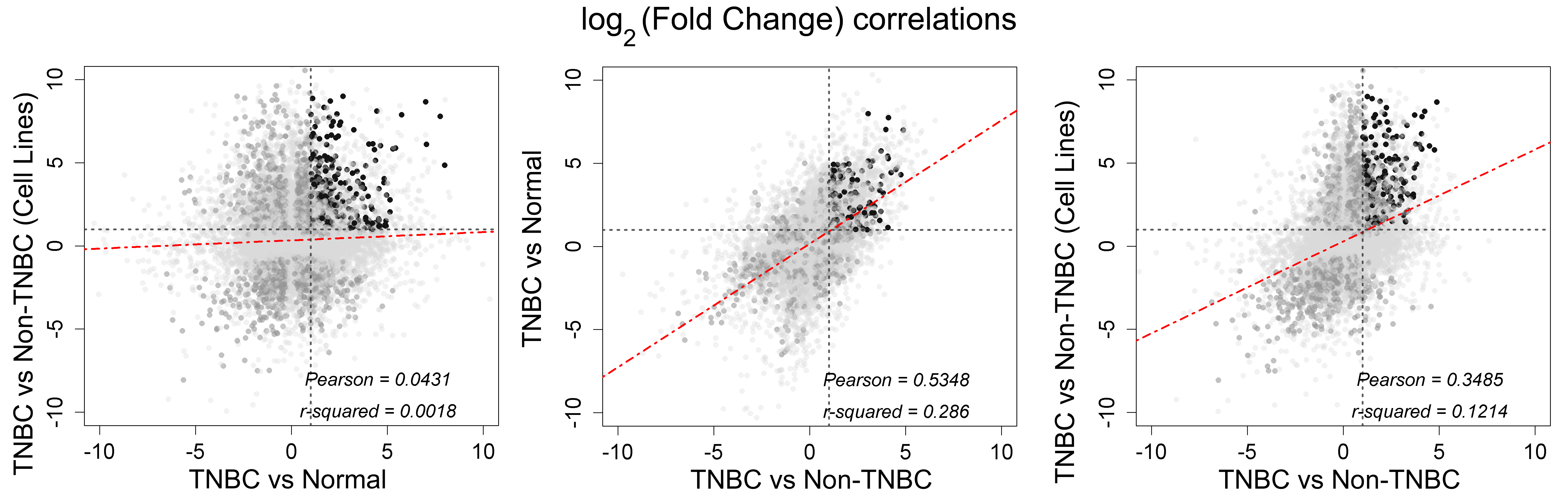

Supplement: Supplementary file 12 — Correlation plots. 2D correlation plots (equivalent to the 2D projections in Fig. 2A) of the FC of DE genes obtained from the comparisons of TNBC versus non-TNBC cell lines and TNBC versus normal tissues (left), TNBC versus normal tissues and TNBC versus non-TNBC tissues (middle), and TNBC versus non-TNBC cell lines and TNBC versus non-TNBC tissues (right) (PNG 1580 kb) [file 12885_2017_3726_MOESM12_ESM.png]

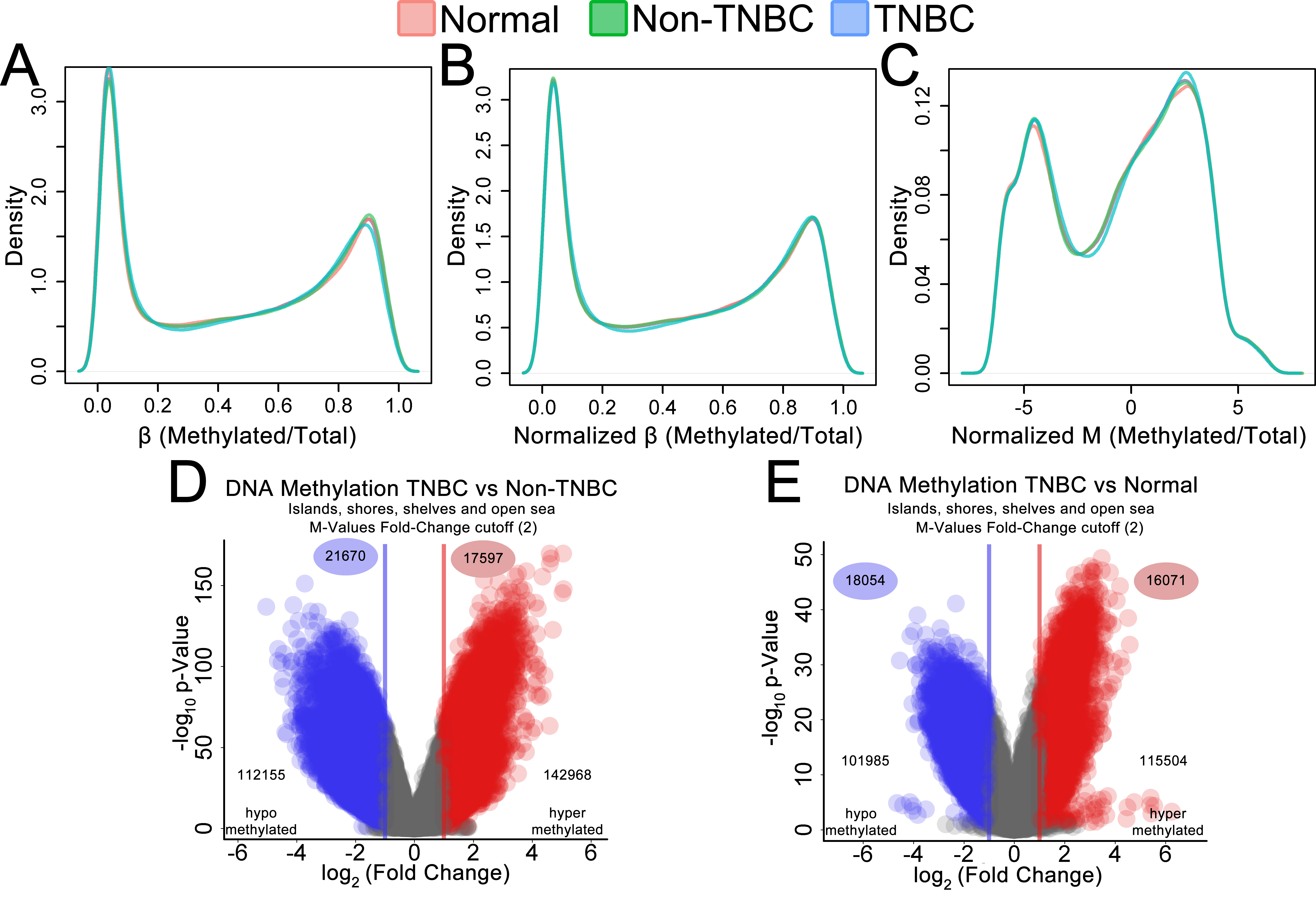

Supplement: Supplementary file 14 — GO analysis of altered pathways in TNBC tissues and cell lines (compared with non-TNBC samples). Biological processes (blue), cellular components (red) and molecular functions (green) equally enriched in TNBC tissues (left) and cell lines (right). These pathways point to events occurring on the membrane, associated with signaling activity and cell motility. Each box denotes 1 order of magnitude of FDR reduction; dashed black lines highlight an FDR = 0.05 (PNG 1759 kb) [file 12885_2017_3726_MOESM14_ESM.png]

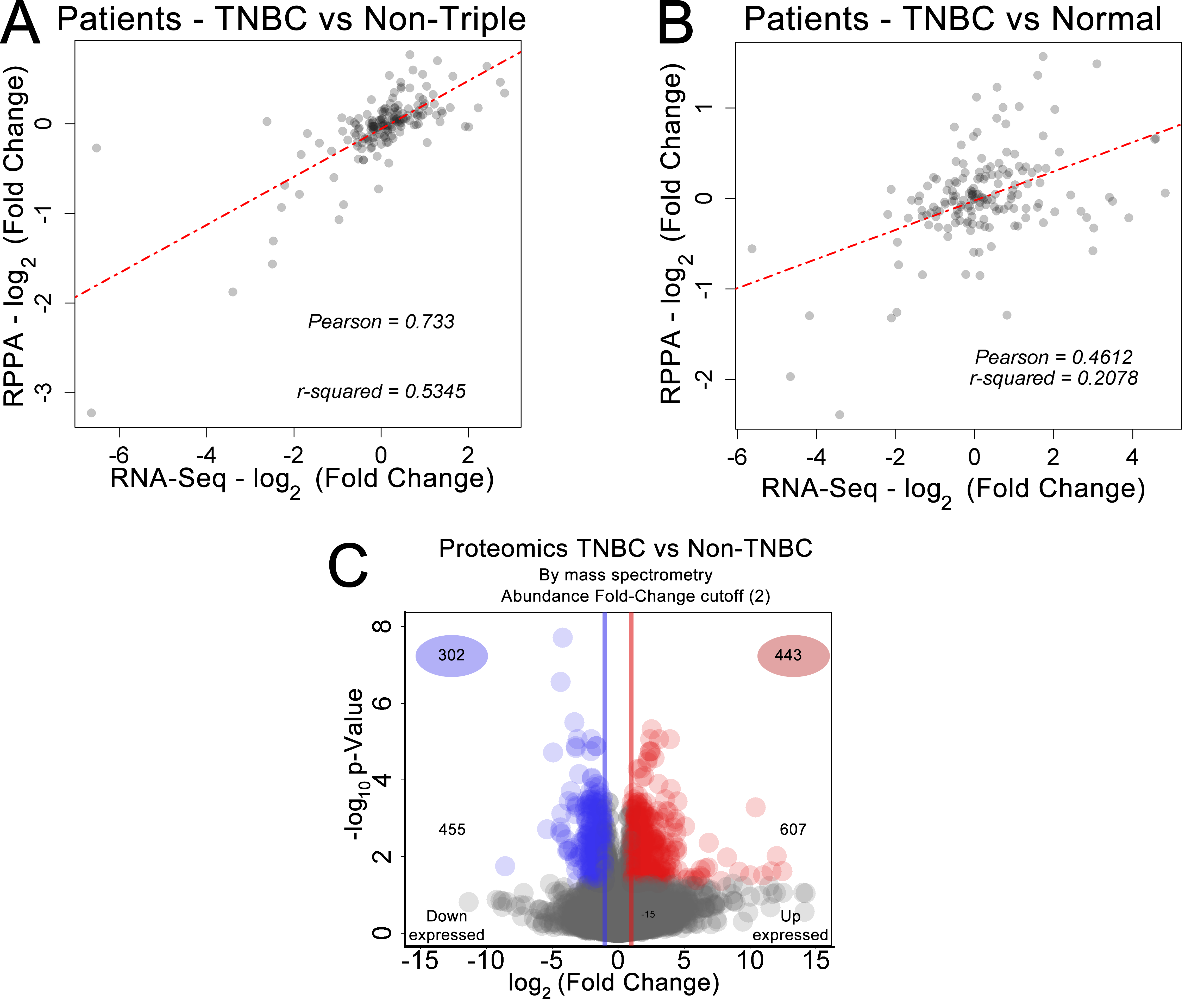

Supplement: Supplementary file 16 — Methylation status of the available probes in the TCGA TNBC x Non-TNBC and normal x TNBC comparisons, as performed by limma (PNG 2076 kb) [file 12885_2017_3726_MOESM16_ESM.png]

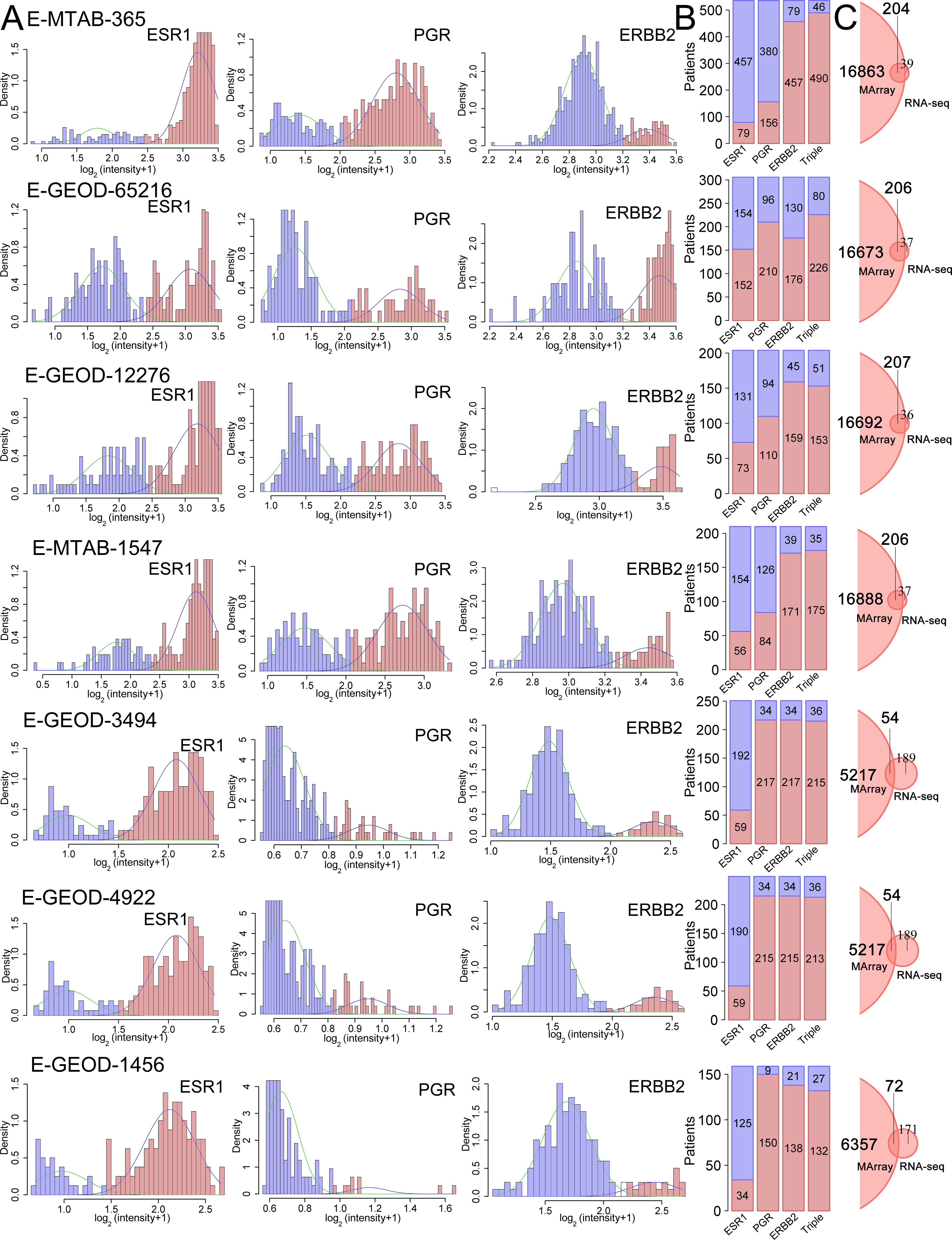

Supplement: Supplementary file 17 — Proteomic analysis of BRCA tissues by using the RPPA and MS data. (A) Comparison between protein level FC (available from RPPA) and mRNA level FC in TNBC vs Non-TNBC (A) and TNBC vs normal (B) comparisons. RPPA data are limited to only 160 proteins. (C) Volcano plot of the FC versus adjusted p values of proteins from MS dataset [64] in TNBC vs Non-TNBC comparison. Non-DE (or DE but with p-value > 0.05) proteins are shown as gray circles, and DE proteins are shown as blue circles when down-regulated and red circles when up-regulated. Proteins with an FC ≥ +2 and an FC ≤ -2, with an FDR < 0.05, were considered up- and down-regulated, respectively. The numbers outside of circles refer to all proteins that passed the FDR cutoff, while the numbers inside of the circles are DE proteins that passed both the FDR and fold-change cutoffs. (PNG 4403 kb) [file 12885_2017_3726_MOESM17_ESM.png]

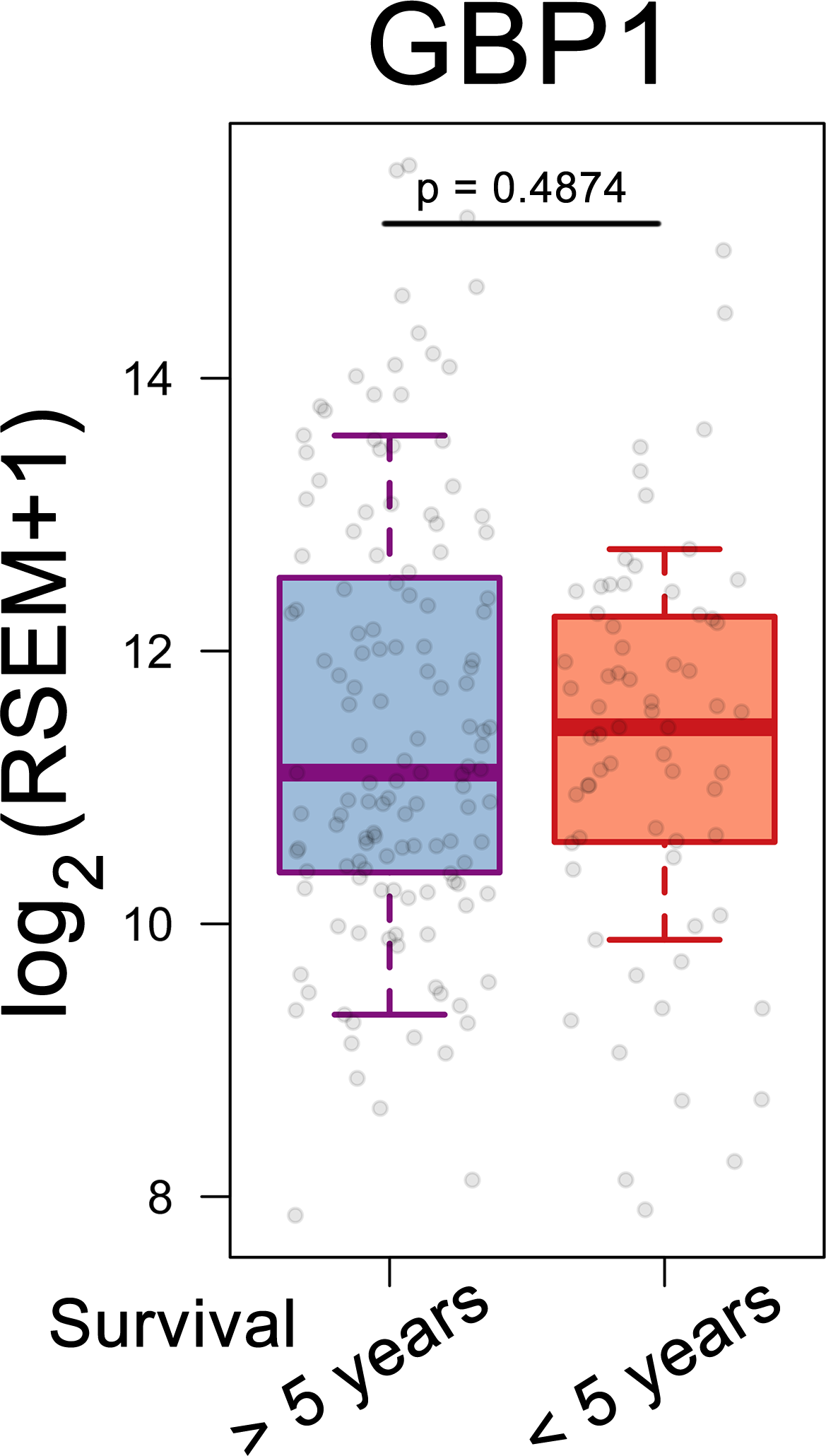

Supplement: Supplementary file 19 — List of genes which expression level impact patients 5 years survival following NCC-AUC analysis (PNG 585 kb) [file 12885_2017_3726_MOESM19_ESM.png]

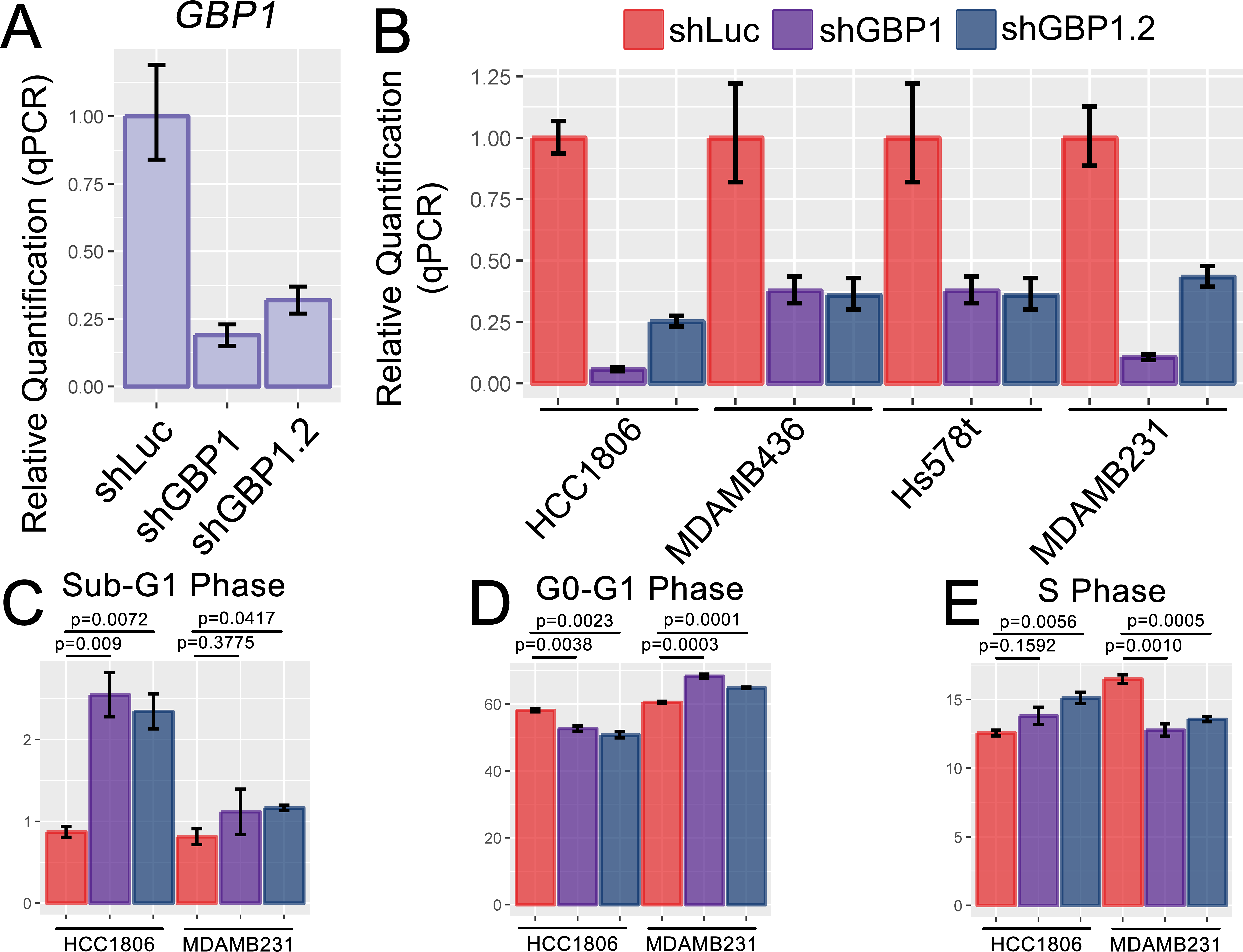

Supplement: Supplementary file 20 — Expression level of GBP1 in patients divided by survival time (more than 5 years survival or less than 5 years survival). The whiskers extend to half of the interquartile range. Gray circles denote each sample. Notches denote the 95% confidence interval of the median. P-Value from Welch’s t-test (PNG 1379 kb) [file 12885_2017_3726_MOESM20_ESM.png]

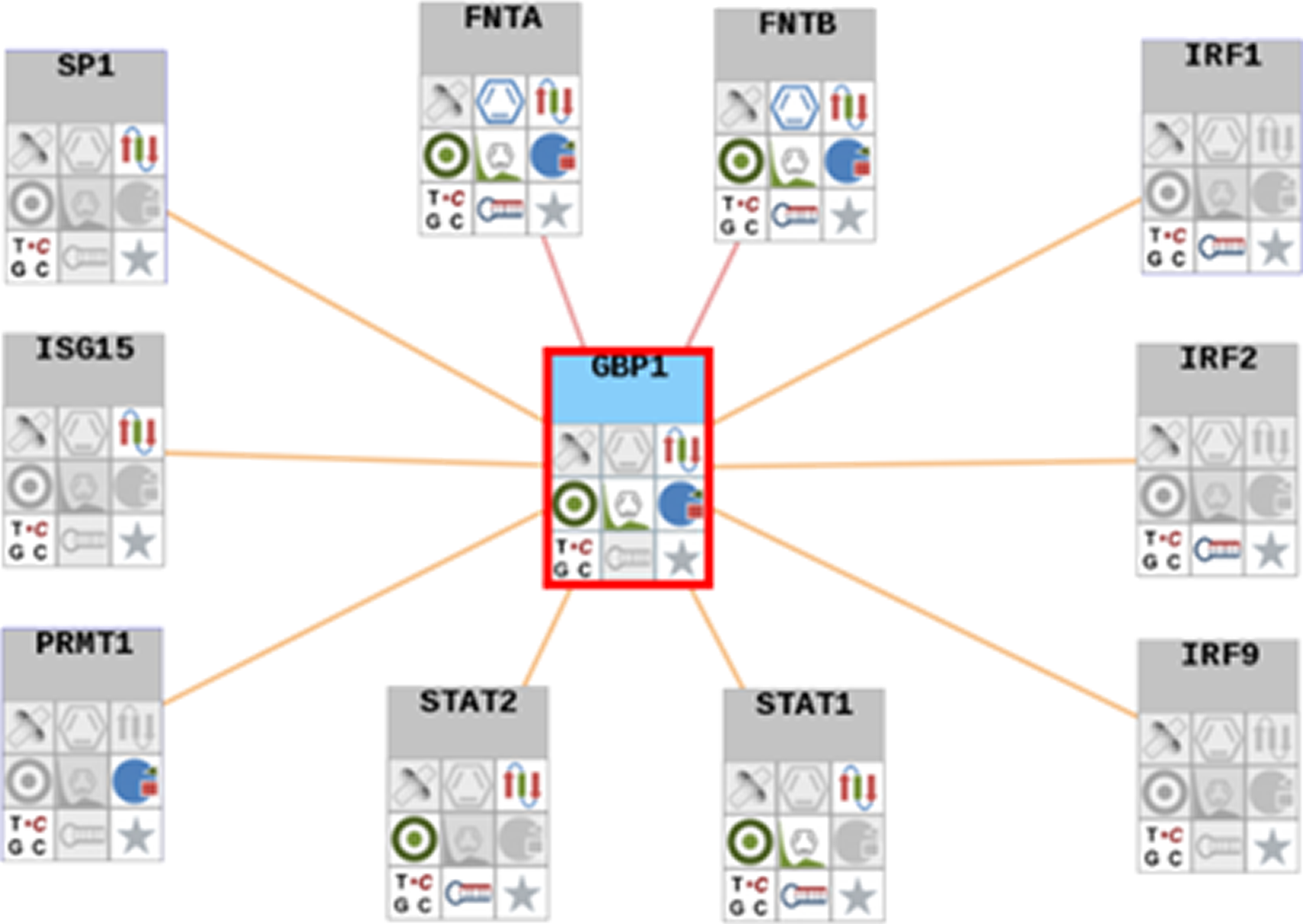

Supplement: Supplementary file 21 — GBP1 knock down evaluation and its effect on cell cycle. (A) qPCR of MDA-MB-231 after GBP1 knock-down, as performed in the end-point assay. (B) qPCR of HCC1806, MDA-MB-231, Hs578t and MDA-MB-231 cells transduced and selected with puromycin to stably express the shRNA sequences. Cell Cycle analysis using DNA content evaluation (as determined by DAPI intensity staining) was executed after imaging attached cells by microscopy. Cells were classified being at the SubG1 (C), G0-G1 (D) or (E) S phase. Error bars represents standard error of the mean. P-Values from Welch’s t-tests (PNG 1679 kb) [file 12885_2017_3726_MOESM21_ESM.png]

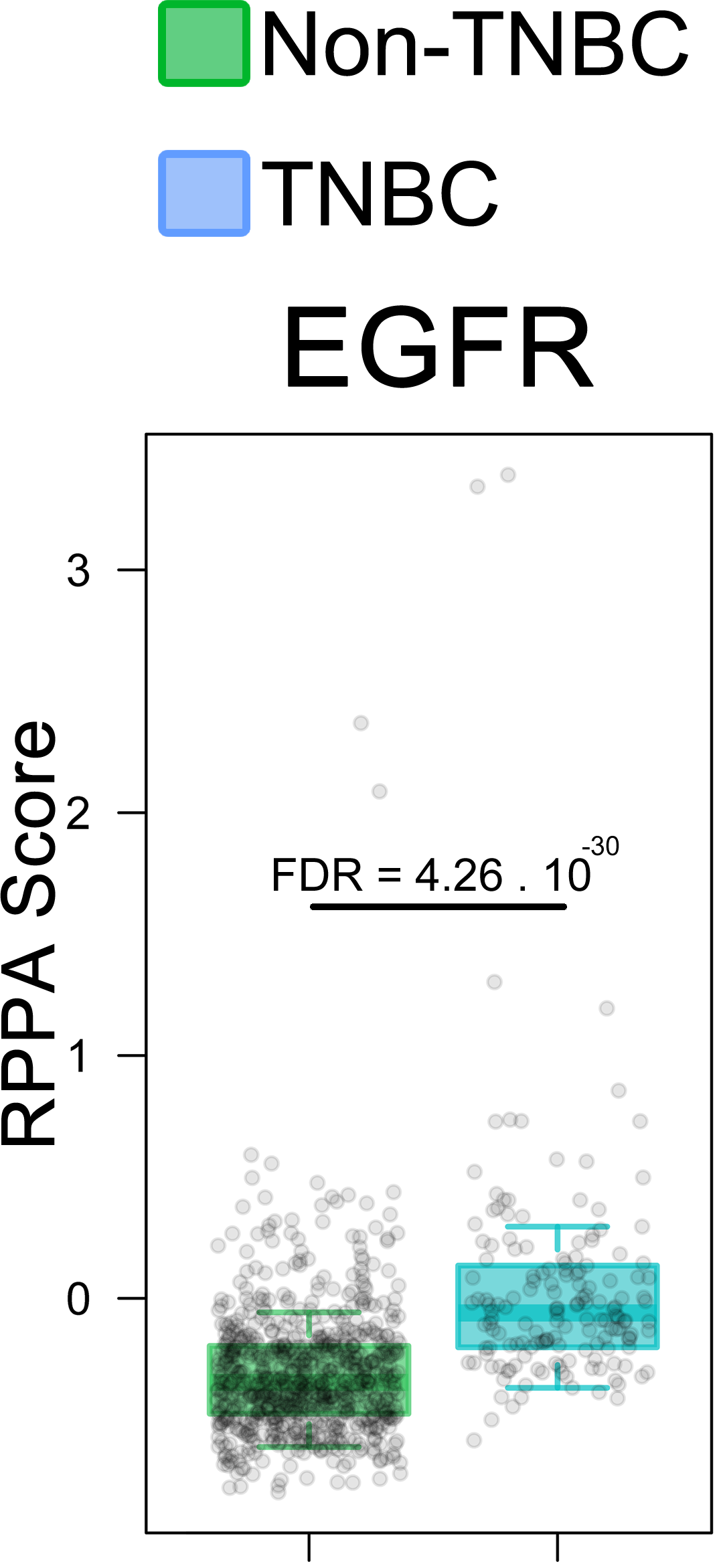

Supplement: Supplementary file 22 — GBP1 Interaction network as defined with the canSAR platform (PNG 724 kb) [file 12885_2017_3726_MOESM22_ESM.png]
